# Dietary Fat Modulation of Gut Microbiota and Impact on Regulatory Pathways Controlling Food Intake

**DOI:** 10.3390/nu15153365

**Published:** 2023-07-28

**Authors:** Sevag Hamamah, Arman Amin, Abdul Latif Al-Kassir, Judith Chuang, Mihai Covasa

**Affiliations:** 1 Department of Basic Medical Sciences, Western University of Health Sciences, College of Osteopathic Medicine, Pomona, CA 91766, USA; sevag.hamamah@westernu.edu (S.H.); arman.amin@westernu.edu (A.A.); abdullatif.alkassir@westernu.edu (A.L.A.-K.); judith.chuang@westernu.edu (J.C.); 2 Department of Biomedical Sciences, College of Medicine and Biological Science, University of Suceava, 720229 Suceava, Romania

**Keywords:** obesity, satiety, neuropeptides, prebiotics, probiotics, synbiotics, weight-loss surgery

## Abstract

Obesity is a multifactorial disease that continues to increase in prevalence worldwide. Emerging evidence has shown that the development of obesity may be influenced by taxonomic shifts in gut microbiota in response to the consumption of dietary fats. Further, these alterations in gut microbiota have been shown to promote important changes in satiation signals including gut hormones (leptin, ghrelin, GLP-1, peptide YY and CCK) and orexigenic and anorexigenic neuropeptides (AgRP, NPY, POMC, CART) that influence hyperphagia and therefore obesity. In this review, we highlight mechanisms by which gut microbiota can influence these satiation signals both locally in the gastrointestinal tract and via microbiota-gut-brain communication. Then, we describe the effects of dietary interventions and associated changes in gut microbiota on satiety signals through microbiota-dependent mechanisms. Lastly, we present microbiota optimizing therapies including prebiotics, probiotics, synbiotics and weight loss surgery that can help restore beneficial gut microbiota by enhancing satiety signals to reduce hyperphagia and subsequent obesity. Overall, a better understanding of the mechanisms by which dietary fats induce taxonomical shifts in gut microbiota and their impact on satiation signaling pathways will help develop more targeted therapeutic interventions in delaying the onset of obesity and in furthering its treatment.

## 1. Introduction

Obesity, characterized by energy imbalance and excess body fat, is a multifactorial disease that has been consistently contributing to worldwide morbidity and mortality over the past several decades [1,2]. It is estimated that the global prevalence of obesity is reaching nearly 650 million individuals with a projection to increase to over one billion by 2030 [3], further predisposing the global population to cardiometabolic disease including but not limited to type 2 diabetes mellitus, cardiovascular disease and metabolic syndrome. Currently, the overall health care costs directed towards prevention, diagnosis and treatment of obesity and related sequelae is two trillion dollars, leading to the creation of the term “globesity” by the World Health Organization [4]. Due to the global burden of disease, a substantial amount of research has been directed towards elucidating the underlying mechanisms behind the pathogenesis of obesity [5]. In recent years, gut microbiota have emerged as potential contributors in the development of metabolic disease through modulation of host epigenetics, promotion of inflammatory states and alteration of satiety signals [6,7]. More specifically, the microbiota-gut-brain axis is thought to regulate these complex processes through bidirectional crosstalk from the enteric nervous system (ENS) and central nervous system (CNS) via vagal afferents, pro-inflammatory cytokines, endocannabinoids, short-chain fatty acids (SCFA) and other microbiota-derived metabolites [8]. Further, dietary nutrients have also been shown to play an intricate role as the main modulator of the microbial composition that impacts modulation of the so-called microbiota gut-brain axis, preceding pathophysiological and phenotypical changes in metabolic disease [9,10]. In general, unhealthy dietary nutrients such as saturated dietary fats increase the Firmicutes/Bacteroidetes ratio [11] that have consistently been associated with many pathological conditions including obesity, T2DM and many other metabolic diseases [12,13]. It has been well documented that high-fat diet induced changes in gut microbiota confer neuronal adaptations that affect central neuropeptides involved in mediating satiety [14]. For example, dietary fats activate orexigenic neuropeptides such as neuropeptide Y (NPY) and Agouti-related protein (AgRP) [15], while also attenuating anorexigenic neuropeptides like proopiomelanocortin (POMC) and cocaine- and amphetamine related transcript (CART) through gut-microbiota dependent mechanisms, contributing to hyperphagia and eventually obesity [16]. Further, it has been shown that gut microbial effects on both leptin expression and body weight were lessened by high fat diets [17]. Dysbiosis of intestinal microbes characterized by increased Firmicutes/Bacteroidetes ratio induced by dietary fats is associated with the elevation of serum ghrelin, a gut hormone that stimulates hunger [18]. Studies have also shown that even a 25% restriction of high-fat diet normalized derangements of ghrelin and leptin as well as AgRP, NPY and POMC expression in the hypothalamus [19]. On the other hand, healthier dietary fats such as omega-3 poly-unsaturated fatty acids (PUFA) have been shown to promote a more favorable gut environment [20], generating lipid mediators that regulate nutrient sensing neuropeptides to combat obesity [21]. Therefore, therapeutic modalities that normalize gut microbiota have been extensively studied including diets such as the Mediterranean diet that is rich in omega-3 PUFA and has been shown to improve the Firmicute/Bacteroides ratio [22]. Additionally, prebiotics, probiotics and synbiotics and even weight loss surgery can also exert therapeutic effects through the optimization of gut microbiota and thereby lessening and even reversing some of the biochemical processes that contribute to the onset of obesity [23,24].

In this review, we present emerging evidence exploring the mechanisms by which dietary fats induce gut microbial changes and how these alterations contribute to hyperphagia and the onset of obesity. As such, we first describe the mechanisms by which gut microbiota interact with gut hormones and neuropeptides to affect energy balance including important metabolites and inflammatory processes. In particular, our review focuses on the reciprocal interaction between gut microbiota and the main satiation hormones, leptin, ghrelin, glucagon like peptide-1 (GLP-1), peptide YY (PYY) and cholecystokinin (CCK), as well as orexigenic and anorexigenic neuropeptides, AgRP, NPY, POMC and CART in control of food intake and regulation of body weight. The effects of dietary interventions on these interactions, particularly by focusing on two main diets with variable opposing health effects, the high fat diet and the Mediterranean diet, will also be discussed. Lastly, we present evidence of how various microbiota altering therapeutic approaches that restore normal gut flora can diminish the deleterious effects associated with obesity.

## 2. Microbiota-Gut Brain Axis in Controlling Food Intake

Over the years, emerging evidence has suggested that gut microbiota play a role in the development of obesity through signaling via the microbiota-gut-brain axis. This so-called microbiota-gut-brain axis serves as a conduit for bidirectional communication between the gut and the brain in which diet-mediated changes in gut microbiota can influence key signaling pathways to affect energy homeostasis [25]. Associated alterations in microbiota-derived neuroactive metabolites may then be transmitted to key areas in the brain that mediate satiation signals, contributing to the onset of hyperphagia [26]. Vagal afferents serve as a major player in this process, by relaying peripheral gut information to hindbrain and hypothalamic neuronal networks that control food intake and regulate energy homeostasis. These vagal terminals reside in gut epithelium within enteric nervous system neurons, in close proximity to gut bacteria, which can directly, or indirectly via its metabolites, influence vagal activity [27]. For example, beneficial bacterial strains such as *Lactobacillus* are shown to activate sensory vagal neurons and increase vagal firing frequency, an effect diminished through vagus nerve ablation in mice [28,29]. Conversely, dietary fat induces the opposite effect in obese mice as demonstrated through the impairment of vagal neural activity when compared to normal weight controls [30]. These findings are supported by studies showing alterations in vagal afferent signaling secondary to high-fat diet feeding, leading to the attenuation of central satiation signals [31]. More specifically, high fat feeding reduces vagal sensitivity to gut mediators such as serotonin and CCK, therefore promoting increased orexigenic receptor activity conducive to hyperphagic behaviors. These effects may directly result from diet-driven dysbiosis which has been shown to have significant associations with vagal remodeling [32]. In addition to vagus-mediated signaling, other important mechanisms that influence satiation signals through gut-brain communication exist, including short-chain fatty acids (SCFA), gut inflammation and endocannabinoids. These mechanisms are intricately related and will be discussed in greater detail in the following subsections.

### 2.1. Short-Chain Fatty Acids and Nutrient Sensing Receptors

Gut microbiota metabolize dietary fibers and resistant starch, which are otherwise indigestible to naturally occurring human enzymes, to yield short-chain fatty acids (SCFA). These SCFA largely consist of acetate, propionate and butyrate, which are the end products of dietary fibers fermentation reactions. The amount of SCFA available in humans is largely dependent on the relative abundances of certain species known to have SCFA-producing capability such as *Bifidobacterium*, *Lactobacillus*, *Faecalibacterium*, *Eubacterium*, *Roseburia*, to name a few. These microbiota-derived metabolites exert a myriad of beneficial effects on host health including enhancing intestinal barrier integrity, as well as reducing systemic inflammation, glucose balance and appetite regulation [33,34,35]. Though the exact mechanisms are not yet fully elucidated, SCFA act on key components within the microbiota-gut-brain axis to exert their benefits. For example, the introduction of microbiota into a germ-free gut environment increased the expression of occludin and claudin-5, two tight junction proteins that decrease blood brain barrier permeability (BBB) [36]. Recent studies have supported the role of SCFA in BBB integrity, with antibiotics increasing its permeability. Inoculation of SCFA-producing *Lactobacillus* and sodium butyrate reverses this effect, again through increasing expression of tight junction proteins [37]. The immunomodulatory role of SCFA is also a significant component in these processes. Butyrate, in particular, has been shown to exert epigenetic influences on gut inflammation through its inherent histone deacetylase inhibitor activity [38]. As such, sodium butyrate decreases neuronal oxidative stress and markers of BBB permeability such as GFAP [39]. These changes were also associated with reduced differentiation of pro-inflammatory cytokines, IL-1β, IL-17A and IL-18 in the striatum and cortex. Therefore, in relative deficient states of SCFA or SCFA-producing bacterial species seen after chronic high-fat diet feeding, neuroinflammation can ensue via increased microglial activity, which are shown to promote synaptic remodeling and neurodegeneration to negatively affect satiation signals [40,41,42]. More specifically, serum triglycerides that cross the BBB contribute to insulin and leptin receptor resistance and impaired satiety [41]. Taken together, these studies show that maintaining healthy concentrations of SCFA is important in the regulatory processes that influence inflammation, satiety and appetite.

Many of the interactions described above are demonstrated to be secondary to SCFA binding to its nutrient sensing receptors. For example, the role of gut microbiota in weight gain is supported by findings showing changes in the expression of gut nutrient-sensing receptors, particularly GPR41 and GPR43 [43,44]. Specifically, intestinal expression of both GPR41 and GPR43 is reduced in germ free mice compared to conventionally raised mice [45]. GPR41 and GPR43 are contained in the adipocytes and colonic epithelium and are activated by SCFA [46] which are involved in weight gain and metabolic homeostasis via GPR43 [47]. For example, mice with overexpression of GPR43 in adipocytes remained lean while being fed a high fat diet by inhibiting fat accumulation in adipocytes [43]. Similarly, GPR43-deficient mice fed a high fat diet develop inflammation and unfavorable remodeling of gut microbial composition with increased Firmicutes and decreased proteobacteria and actinobacteria [43]. These changes showing elevated inflammatory markers like tumor necrosis alpha (TNF-α) and increased Firmicutes/Bacteroidetes ratio are consistent with changes that precede progression, and promote susceptibility, to developing obese phenotype [48]. Lu et al., support these findings by showing that the expression of GPR41 and GPR43 was associated with a reduction in Firmicutes and an increase in Bacteroidetes [47]. At the same time, this resulting increase of GPR41 and GPR43 expression from SCFA administration was found to limit chronic inflammation while enhancing triglyceride hydrolysis and free fatty acid oxidation to reduce body weight [47]. Therefore, both GPR41 and GPR43 receptors play an important role in nutrient sensing and energy balance by protecting against weight gain in response to dietary fat-induced changes in gut microbiota and SCFA.

### 2.2. High-Fat Diet, Gut Bacteria, Intestinal Permeability, and Inflammation

Gut and brain inflammation is intricately linked through the microbiota-gut-brain axis contributing significantly to altered satiation signals seen in obesity and hyperphagia. In states of dysbiosis induced by high-fat diets, gram-negative bacteria predominate, which naturally contain lipopolysaccharides (LPS) within their outer membranes. LPS, in turn, bind toll-like receptor 4 (TLR-4), promoting inflammation via secretion of pro-inflammatory cytokines such as TNF-α, IL-1 and IL-17. These inflammatory factors attenuate vagal mediated feedback inhibition of food intake by acting on vagal afferents [49]. Further, LPS and its downstream effects promote local inflammation in the gut increasing intestinal barrier permeability. LPS may translocate across the gut barrier into the systemic circulation to exert widespread inflammatory cascade, with sustained increases in LPS, leading to systemic inflammation and hyperphagic states [50].

Interestingly, it has also been shown that the nodose ganglion (NG), where the cell bodies of vagal afferents reside, is an important location mediating immune responses by gut microbiota and satiation signals [51]. For example, high-fat diet associated bacterial species promote inflammation in this region, as measured through increases in IL-6, interferon-gamma and TNF-α gene expression, as well as chemokine ligands which are important in cell-to-cell communication [52]. Likewise, byproducts of gram-positive bacteria such as lipoteichoic acid (LTA), which is functionally equivalent to LPS, represent a source for NG vagal inflammation. Further, it has been shown that low-grade inflammation via increased nitric oxide synthase (iNOS) expression, similar to ones seen in obesity, influences energy intake [53]. This was demonstrated through administration of an iNOS inhibitor, which reduced food intake and decreased body weight gain, by increasing NG neuronal excitability through potassium channel leak currents. Importantly, many gut hormone and neuroactive peptide mediators including leptin, glucagon, CCK, peptide YY, GLP-1, and NPY act at the NG to evoke post-ingestive satiety effects [54]. For example, high-fat diets impair vagal sensitivity through increasing the expression of leptin inhibitors like suppressor of cytokine signaling 3 and protein tyrosine phosphate 1B (PTP1B) in the NG [55]. Further, glucagon interaction with its receptor activates NG vagal afferents as observed through glucagon induced phosphorylation of ERK1/2 and resulting increases in calcium concentrations [56]. Taken together, these findings provide an important link between gut microbiota, high-fat diets, and associated effects on inflammatory pathways that influence key gut peptides with the role in control of food intake and energy regulation.

### 2.3. Endocannabinoids, Gut Microbiota, and High-Fat Diets

There has been strong evidence for the role of endocannabinoids (ECB), a group of naturally occurring lipid neurotransmitters, and their receptors in regulating food intake. In adequate concentrations, endocannabinoid ligands such as anandamide and 2-aracdonoylglycerol (2-AG) have positive effects on brain development, though excessive activation of the ECB/cannabinoid receptor systems have been associated with functional antagonism in related brain circuits leading to dysregulated energy balance and obesity [57]. For example, high fat diets have been shown to significantly increase endocannabinoids, both centrally and peripherally, influencing hypothalamic appetite regulatory neuronal pathways [58]. Excessive ECB and cannabinoid receptor type 1 (CB1) signaling promote dysfunctional episodic memory as well as reduced synaptic plasticity and neurogenesis in the mice hippocampus [59]. Therefore, HFD-induced increases in ECB may hamper the cognitive control of feeding behaviors, which have been heavily implicated in both food choice and weight gain [60].

Further, ghrelin and anandamide cause changes in the ECB gastrointestinal system by altering the sensitivity of vagal afferents and signaling via the microbiota-gut-brain axis [61]. As such, HFD-induced obesity increased CB1, ghrelin receptor and fatty acid amide hydrolase (FAAH) mRNA content in the NG, contributing to a reduction of vagal afferent sensitivity. It should also be noted that the modulatory influence of ECB on vagal afferent signaling is dose-dependent under standard diets with dual excitatory and inhibitory effects shown on stretch-receptors [62]. Interestingly, HFD-induced obesity causes a dominant inhibitory effect on gastric vagal afferents independent of ECB concentration. However, inhibition of gastric vagal afferents is thought to be mediated through ghrelin, which may decrease ECB-induced stretch sensitivity [63]. Ghrelin is also reduced in HFD-induced obesity, again contributing to altered satiation signals controlled by the ECB-CB1 system.

In addition to directly influencing neuronal circuits, endocannabinoid and LPS levels have been shown to be positively correlated [58]. Specifically, LPS promotes synthesis of anandamide, an endogenous lipid ligand of CB1 receptors, within macrophages which activate NF-KB immune and inflammatory cascades [64]. LPS also inhibits FAAH, the enzymes that degrade ECB including anandamide [64]. Therefore, gram-negative enteric bacterial overgrowth seen commonly in HFD feeding can potentiate the role of the ECB/CB1 system to compromise gut barrier integrity and promote inflammatory sequelae. Interestingly, a recent study has shown that the ECB/CB2 system is influenced by exercise, which decreases LPS-induced neuroinflammation in the rat hippocampus [65]. These findings were associated with decreased CB2 expression, providing further insight into the role of ECB in obesity and potential therapeutic methods that target its receptors. This provides strong evidence supporting the effects of excessive cannabinoid receptor activation and LPS-induced local and systemic inflammation in producing unfavorable changes in HFD-induced obesity.

## 3. Microbiota and Satiation Signals

Several studies have shown that gut microbiota dependent mechanisms play an important role in decreased sensitivity to both peripheral and central satiation signals in high fat-induced obesity. In this regard, gut microbiota have been found to modulate the activity of both gut peptides such as ghrelin, leptin, and CCK as well as orexigenic and anorexigenic neuropeptides like AgRP, NPY, POMC and CART. Neurons expressing these neuropeptides are responsive to changes in gut peptides, particularly leptin but also ghrelin and insulin.

### 3.1. Gut Microbiota and Its Effects on Leptin and Ghrelin

Leptin is a non-glycosylated hormone synthesized and secreted from adipose tissue that serves to inhibit hunger and hyperphagia by regulating hypothalamic signaling [66]. More specifically, leptin directly influences the secretion patterns of orexigenic peptides, NPY and AgRP, as well as anorexigenic peptides, POMC and CART, by inhibiting or activating neurons by binding to leptin receptors in the arcuate nucleus of the hypothalamus [67]. Therefore, states of leptin resistance play an important role in balancing the effects of these gut peptides to regulate body weight. The reason for leptin resistance is unknown, although there are several known contributing factors that can be attributed to high-fat diet-induced obesity including increased inflammatory signaling, accumulation of lipid metabolites, neuroendocrine axis dysfunction and excessive leptin exposure [68,69]. Interestingly, impairment in leptin signaling is also attributed to the changes that dietary fats induce in the gut microbiota. For example, germ-free mice showed increased leptin expression at baseline and hypermethylation of the leptin promoter under high fat diet feeding, as compared to conventionally raised mice [17]. This indicates that normal fat diet-fed mice with normal gut microbiota are most responsive to exogenous leptin, while HFD-induced hypermethylation of the leptin promoter can confer resistance.

**Figure 1:**
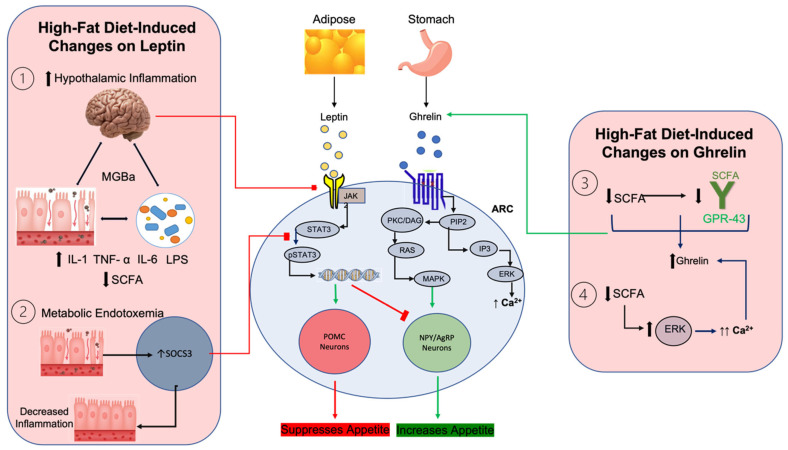
High fat diet-induced changes in leptin and ghrelin and resultant effects on satiation signaling pathways and central neuropeptides. Leptin binds to its receptor, initiating signaling via the JAK/STAT3 pathway which leads to increased stimulation of POMC and inhibition of NPY/AgRP neurons. Ghrelin binds to its receptor (GHSR-1a) initiating signaling through the PKC/DAG, RAS and MAPK pathway eventually leading to stimulation of NPY/AgRP neurons. Ghrelin also signals via the ERK pathway to increase calcium, which is involved in feedback mechanisms that regulate ghrelin release. (1) High-fat diets induce hypothalamic inflammation, which is associated with an increase in pro-inflammatory cytokines, LPS secretion and leptin resistance. (2) High-fat diets induce metabolic endotoxemia, leading to increased SOCS3, which inhibits STAT3 phosphorylation. (3) High-fat diets decrease SCFA binding to GPR-43, which leads to increased ghrelin concentrations. (4) Decreased SCFA induced by high-fat diets increases ERK and calcium which further stimulates ghrelin secretion. Abbreviations: MGBa, microbiota-gut-brain axis; IL-1, interleukin-1; TNF, tumor necrosis factor; IL-6, interleukin-6; LPS, lipopolysaccharides; SOCS3, suppressor of cytokine signaling 3; STAT3, signal transducer and activator of transcription 3; pSTAT3, phosphorylated signal transducer and activator of transcription 3; POMC, pro-opiomelanocortin; PKC, protein kinase C; DAG, diacylglycerol; MAPK, mitogen activated protein kinase; PIP2, phosphatidylinositol 4,5-bisphosphanate; IP3, inositol 1,4,5-triphosphate; ERK, extracellular signal-related kinase; NPY, neuropeptide Y; AgRP, agouti-related peptide; Ca^2+^, calcium; ARC, arcuate nucleus; SCFA, short-chain fatty acids; GPR-43, G-coupled protein receptor 43.

In addition, dietary fat-induced changes in the gut microbiota have been involved in leptin resistance via hypothalamic inflammation and suppressor of cytokine signaling 3 (SOCS3). SOCS3 is an inhibitor of STAT3, an important marker of leptin signaling as deletion of the STAT3 gene in the CNS induces obesity [70] (Figure 1). Reduced SOCS3 expression in adipose tissue is protective against high-fat diet induced obesity and insulin resistance [71]. It has also been shown that SOCS3 knockout mice had improved leptin sensitivity through increased hypothalamic STAT3 phosphorylation and POMC induction, while showing resistance to high-fat diet-induced obesity [72]. By contrast, saturated fat diets have been shown to increase levels of SOCS3 as well as TNF-α, both of which can contribute to obese phenotype [73]. However, the former study uses a murine model, while the latter shows findings in human subjects which may contribute to the observed differences. Additionally, concentrations of SOCS3 are increased in conventionally raised mice versus germ-free mice [16]; therefore, leptin resistance can be mediated by gut microbiota, particularly in states of inflammation. Though it can confer leptin resistance via inhibiting STAT3 activation, SOCS3 serves important roles in reducing severe systemic inflammation associated with high-fat diet induced dysbiosis [74]. For example, the introduction of probiotic species like *Lactobacillus rhamnosus GG* induced increased IL-10R activation, an anti-inflammatory cytokine receptor, mediated regulation of inflammatory pathways resulting from gut dysbiosis [75]. *Lactobacillus rhamnosus* also increases STAT3-phosphoryaltion, causing an induction of SOCS3 as well. Taken together, these findings suggest that states of metabolic endotoxemia and inflammation induced by high-fat diets lead to increased concentrations of SOCS3, thereby leading to leptin resistance and contributing to hyperphagia and obesity.

Hypothalamic inflammation also plays a significant role in promoting leptin resistance and is influenced by high-fat diet-induced changes in gut microbiota concentrations (Figure 1). Excessive microglial and astrocyte activation along with increased concentrations of inflammatory mediators in the hypothalamus after high-fat diet feeding is associated with leptin resistance and precedes weight gain [76]. On the other hand, hypothalamic microglial depletion via diphtheria toxin introduction eliminates saturated fat-induced neuroinflammation and enhances leptin signaling and reduced food intake [77]. Further, the presence of gut microbiota has been found to promote changes in microglial gene expression, which contribute to their maturation and activation, while germ-free mice show diminished amounts of reactive oxygen species and microglial dysfunction [78]. These findings can be explained by microbiota derived SCFA, which activates GPR43 to promote microglial maturation as evidenced through GPR-43 knockout murine models that have immature and defective microglia [79]. Interestingly, recent findings have shown that a Glucagon-like peptide 1 receptor (GLP-1R) dependent pathway may be responsible for the decreased hypothalamic inflammation observed in germ-free mice as these mice have increased GLP-1 concentrations [80]. It was also found that the inhibition of GLP-1R induces microgliosis and therefore negatively affects leptin sensitivity. Further, another recent study showed that dysbiosis of gut microbiota after high-fat diet feeding and characterized by elevations of harmful families of bacteria such as *Rumminococcacae* and *Lachnospiraceae* associated with metabolic endotoxemia, impair daily secretions of GLP-1 [81]. As such, the presence of certain gut bacteria influences hypothalamic inflammation through mechanisms that include reducing GLP-1 levels and activating nutrient sensing receptors like GPR-43, causing changes in leptin sensitivity and feeding patterns. It is also important to note that gliosis after high-fat diet feeding may be reversible as evidenced through immunohistochemical and magnetic resonance imaging techniques in rodent models [82]. This reveals some of the mechanisms by which microbiota-derived therapeutic interventions can improve leptin sensitivity, which will be discussed later.

In contrast to leptin, ghrelin is an orexigenic gut hormone released mainly from the stomach and duodenum that increases in fasting states and increases hyperphagic drive, therefore serving as another peripheral signaling molecule that relays key information regarding energy status [83]. Additionally, study findings have shown that increases in SCFA production may reduce serum ghrelin concentrations [84] (Figure 1). There are two mechanisms proposed that may explain these findings. First, it has been shown that SCFA binding to its nutrient sensing receptor, GPR43, diminishes ghrelin secretion by exerting G-alpha-inhibitory downstream effects. Diets enriched in saturated fats generally promote unfavorable changes in gut microbiota, and overgrowth of non-SCFA producing bacteria. Therefore, in states of depleted SCFA secondary to high-fat diet induced obesity, ghrelin levels can increase due to decreased GPR43-mediated inhibition of ghrelin secretion. In contrast, consumption of beneficial omega-3 PUFA, which increases favorable SCFA-producing bacteria, decreases fasting ghrelin and increases fasting peptide YY [85]. Second, it has been shown that *Lactobacillus* and *Bifidobacterium*, prolific SCFA-producing genera, attenuate ghrelin receptor (GHSR-1a) signaling [86]. Microbiota-derived SCFA accomplish this by preventing ghrelin-mediated calcium influx into cells and increasing ERK1/2-mediated phosphorylation, an important downstream kinase that mediates GHSR-1a effects. Though the mechanisms for increased ERK1/2 are unclear, one study has shown important concentration-dependent effects of calcium and the role of calcium sensing receptors in ghrelinergic activity [87]. The introduction of low amounts of calcium resulted in an inhibitory effect of CaSR on ghrelin secretion [87]. These findings suggest that microbiota may exert their effects on ghrelin secretion through influencing GPR-43 and GHSR-1a activity as well as through calcium mobilization.

### 3.2. Enteroendocrine Signaling, GLP-1, PYY, and Gut Microbiota

**Figure 2:**
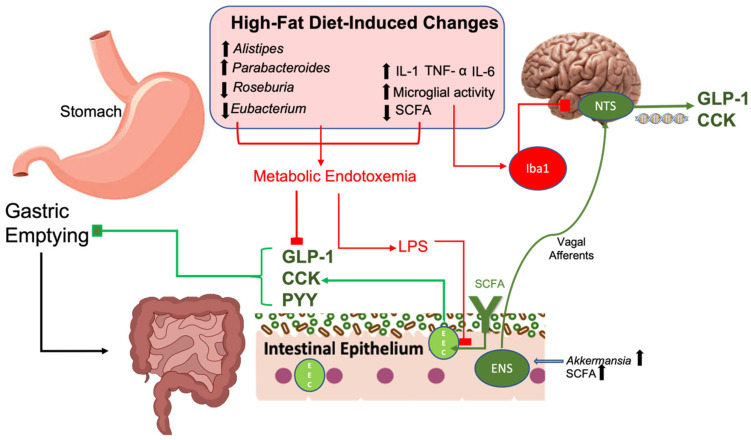
High fat diet-induced changes in GLP-1, CCK and PYY and subsequent effects on gut microbiota and satiation signals. High-fat diet promotes increases in *Alistipes* and *Parabacteroides* and decreases in SCFA-producing species like *Roseburia* and *Eubacterium*. Concurrently, high-fat diets increase pro-inflammatory cytokines, microglial activity measured by Iba1, which inhibit GLP-1 and CCK activity in the NTS. In the gut, this combination promotes metabolic endotoxemia through increases in LPS, which inhibit satiation through GLP-1, CCK and PYY through reductions in gastric emptying. Abbreviations: GLP-1, glucagon-like peptide 1; CCK, cholecystokinin; PYY, peptide YY; LPS, lipopolysaccharides; SCFA, short-chain fatty acids; EEC, enteroendocrine cells; NTS, nucleus of solitary tract; ENS, enteric nervous system; IL-1, interleukin-1; TNF, tumor necrosis factor; IL-6, interleukin-6; Iba1, ionized calcium binding adaptor molecule-1.

Enteroendocrine signaling serves as another important mechanism by which dietary fat and its resulting changes in gut microbiota influence gut hormones [88]. Within the epithelium of the GI tract, specialized endoderm-derived enteroendocrine cells (EECs) reside and secrete CCK, GLP-1 and PYY in response to nutritional status and microbiota-derived metabolites [89]. For example, SCFAs, which are detected by nutrient sensing receptors, influence the release of PYY, an anorexigenic gut hormone, in the distal intestine. Circulating concentrations of PYY are increased postprandially and decreased by fasting, with proportional increases as a function of SCFA concentrations [90]. Similarly, recent findings in mouse studies have shown that taxonomical shifts in the gut microbiota after a high-fat diet aggravate PYY signaling through increasing the abundance of non-SCFA and inflammatory microbial genera such as *Alistipes* and *Parabacteroides* [91,92] (Figure 2). These findings were associated with alterations in tight junction proteins, indicating diet-dependent interactions between gut microbiota and enteroendocrine signaling. Interestingly, microbiota transfer from healthy human donors also increased densities of enteroendocrine cells in the colon, resulting in notable increases in PYY [93].

GLP-1 is another important gut hormone that promotes satiety and is released after the ingestion of fats and carbohydrates [94]. Recently, GLP-1 agonists have become one of the first-line pharmacological treatments for obesity due to their potent ability to reduce gastric emptying, glucagon secretion and weight gain [95]. GLP-1 is rapidly degraded after secretion and therefore can act locally on the enteric nervous system as well as vagal afferents to exert its effects [94]. Increasing evidence has shown that its release is intricately related with gut microbiota. For example, *Akkermansia muciniphilia* introduction in mice fed a high-fat diet promotes systemic GLP-1 secretion by inducing thermogenesis in brown adipose tissue [96]. Namely, *Akkermansia* secreted protein, P9, was responsible for GLP-1 secretion through its interaction with intracellular adhesion molecule-2, an effect which was diminished by the pro-inflammatory cytokine interleukin-6 that is increased in chronic high fat feeding and obesity in mice [96,97]. By contrast, low carbohydrate and low-fat diets exert opposite effects by promoting higher abundances of SCFA-producing bacteria such as *Eubacterium* and *Roseburia*, with associated and sustained increases in GLP-1 concentrations, an effect observed in human subjects with type 2 diabetes mellitus [98]. Taken together, these findings provide strong evidence for the detrimental role that high fat diets have on enteroendocrine signaling, specifically through their effects on microbiota and their metabolites.

### 3.3. CCK, Gut Microbiota, and Obesity

Cholecystokinin (CCK), a gut hormone involved in mediating gastric emptying and digestive processes, regulates food intake and HFD-induced weight gain through vagal dependent mechanisms [99]. Vagal afferents innervating the gut convey peripheral signals to the brainstem, particularly to the nucleus of solitary tract (NTS). Diet-induced obesity through feeding a high-fat diet in rodent models has shown decreased post-prandial neuronal activation in the brainstem [100]. More specifically, high fat feeding-induced microbiota changes reduce NTS vagal afferent fibers activation [101]. More specifically, immunocompetent ionized calcium binding adaptor molecule-1 (Iba1) positive cells were present in NG, contributing to decreased innervation in the medial NTS with associated loss of satiety via reductions in CCK. Iba1 cells are a marker of microglia activity, which are resident macrophages of the CNS and their excess activity can cause significant neuroinflammation and neuronal damage [102]. The reverse has also been shown to be true when feeding healthy diets, such as a high-protein diet, which led to increased vagal sensitivity to CCK in mice with diet-induced obesity [103]. These findings were associated with increased *Akkermansia*, a gut microbial genus, found to decrease intestinal permeability through increases in occludin [104]. Additionally, it is believed that gut microbiota further contributes to this process by producing SCFA to mediate CCK gene expression [105]. It is shown that gut microbiota-derived SCFA can act directly on, and activate, vagal afferent neurons [106]. This is supported by findings showing that germ free mice have decreased intestinal expression of CCK [45]. However, it is not just the presence of gut microbiota that facilitates adequate CCK signaling, but rather a favorable gut microbial composition with beneficial bacterial species. High-fat diet-induced obesity is shown to promote dysbiosis leading to a state of systemic inflammation that induces hyperphagia by altering vagally mediated satiety via CCK [107]. For example, feeding potato-resistant starch reversed the blunted CCK signaling initially induced by a HFD through improving microbial dysbiosis, increasing fecal SCFA and vagal function [107]. It is also important to note that the activity of CCK is augmented through activation of leptin receptor expressor cells in the hypothalamus [108]. In particular, it was found that leptin only enhanced the CCK response in rats that were fed a high fat diet but did not exaggerate CCK activity in rats fed a diet with low fat content. Therefore, it is important to consider the mechanisms by which a high fat diet attenuates leptin signaling as described in prior sections as it may have a significant role in the blunted CCK activity commonly seen in HFD-induced obesity. Taken together, these findings suggest that diet-induced obesity through the introduction of HFD impairs CCK signaling through gut microbiota dependent mechanisms, which can alter feeding patterns leading to hyperphagia and obesity.

### 3.4. Gut Microbiota and Its Effects on Orexigenic Neuropeptides

Appetite increasing neuropeptides, NPY and AgRP, are contained within the arcuate nucleus of the hypothalamus and their activities are controlled by feedback mechanisms. Hypothalamic neurons co-expressing NPY/AgRP detect changes within the gut and respond to signals from ghrelin, leptin and insulin to promote energy homeostasis. The importance of these neurons in mediating satiety is shown through studies that ablate NPY/AgRP neurons causing reduced feeding behaviors [109], while stimulating these same neurons induces hyperphagia [110] and food seeking [111]. High fat feeding-induced obesity affects the NPY/AgRP co-expressing neurons in the arcuate nucleus via cytokines that promote hypothalamic inflammation and astrocytosis [112]. Important mediators of these processes include Inhibitor of KB kinase-beta, SOSC3, c-Jun N-terminal kinases (JNK) and protein kinase C, which contribute to obesity via leptin resistance in AgRP neurons [113]. More specifically, study findings have shown that high fat diet feeding upregulates SOCS3, which first affects the activity of AgRP neurons prior to other neuropeptides to induce leptin resistance [114], indicating the important role of these neurons in responding to short-term changes in dietary fat intake. Gut microbiota are also found to contribute to these processes. We have recently shown that gut microbiota can normalize levels of the orexigenic neuropeptides, NPY and AgRP, in germ-free mice through leptin-dependent mechanisms [115]. More specifically, conventionalization of germ-free mice after 10 days decreased mRNA expression of AgRP and NPY in the hypothalamus similar to that of conventionally raised mice. These findings may be attributed to the presence of microbiota metabolites, particularly SCFA which have been shown to modulate GABA signaling in the arcuate nucleus of the hypothalamus [116].

Additionally, several specific bacterial species have been associated with increased hypothalamic NPY mRNA concentrations [117]. During food restriction, several species of *Lactobacillus* spp. and *Clostridium cocleatum* were elevated, while *Burkholderiales* decreased, a finding correlated with changes in NPY. Study findings have also identified *Lactobacillus* as being decreased after high-fat diet feeding, a genus associated with anti-obesity effects through decreasing inflammation and preventing fat accumulation [118]. Again, positive changes induced by *Lactobacillus* spp. can be attributed to their production of SCFA, which alter signaling in key hypothalamic regions associated with NPY and AgRP, as mentioned earlier. These positive effects on NPY and AgRP associated with *Lactobacillus* spp. may also stem from its effects on the reduction of gram-negative species such as *Bacteroides* and *Desulfovibrio* spp., and are characteristically elevated after chronic HFD feeding [118]. Likewise, NPY and AgRP response to glucose is blunted by LPS produced from gram-negative bacteria [119]. Recent studies have supported this notion by showing that high fat induced changes in gut microbiota, including increases in Actinobacteria and Firmicutes/Bacteroidetes ratio that were also associated with similar changes in NPY. Taken together, there is strong evidence for the modulation of orexigenic neuropeptides via HFD-induced alterations in gut microbiota, their metabolites, and the hypothalamic regions where they exert their effects.

### 3.5. Gut Microbiota and Effects on POMC and CART

Appetite decreasing neuropeptides, such as POMC and CART, are found primarily in the arcuate nucleus neurons but also other areas throughout the hypothalamus [120]. Approximately 35–70% of POMC and CART neurons contain leptin receptors and are therefore leptin responsive, with leptin-deficient mice showing reduced CART mRNA expression [120,121]. Activation of POMC neurons via leptin promotes cleavage of POMC for the formation of alpha-melanocyte-stimulating hormone (alpha-MSH), which mediates anorexigenic effects by binding melanocortin-4-receptor (MC4R) [122]. As such, hypothalamic inflammation increased expression of SOCS3 and other leptin altering mechanisms induced by high-fat diets can also influence POMC and CART neurons, contributing to changes in eating behaviors. Further, an overstimulation of the endocannabinoid system in dietary fat-induced obesity may also contribute to diminished leptin response in POMC neurons [123]. Peripheral treatment with a CB1R antagonist improved leptin sensitivity through activation of MC4R-mediated signaling to elicit hypophagia. In addition, POMC neurons have been shown to express CB1R on their plasma membranes [124]. Therefore, significant increases in endocannabinoids such as 2-arachidonoylglycerol production activates ERK1/2, which inhibits STAT3, thus suppressing POMC gene transcription and the effects of leptin. It is also important to note that high-fat diet-induced changes in gut microbiota such as reductions in *Parasuterella* spp. can also be dependent on genetic factors such as polymorphisms in MC4R in obese humans [125]. Therefore, a combination of environmental and genetic factors may contribute to worsening hypothalamic inflammation, endocannabinoid dysregulation, and microbiota-gut-brain disruptions in response to the consumption of dietary fats.

## 4. Dietary Interventions and Satiation Signals

In recent years, significant research has uncovered the multitude of effects that dietary interventions exert on gut microbial composition. Multiple rodent studies have shown that a high fat diet induces significant changes in gut microbiota [126,127], most notably with an increase in the ratio of Firmicutes/Bacteroides phylum. An increase in Firmicutes, a gram-positive bacterium, is associated with a predisposition to increased body fat [48]. Further, several studies have shown that changes in gut microbiome at low taxonomic levels are more variable following consumption of a high fat diet [127], which has been attributed to the types of dietary fat. For example, a diet high in saturated fatty acids leads to a reduction in microbiota diversity, richness and Bacteroidetes. On the other hand, a diet rich in unsaturated fatty acids such as fish oil, leads to the opposite effect, with observed increases in microbiome diversity, richness and Bacteroidetes. These changes and the effects on satiation signals will be discussed in detail in the following sections.

### 4.1. Saturated Fats

Multiple interrelated mechanisms have been proposed to explain the association between dietary saturated fats and inflammatory conditions such as obesity, including its role in the modulation of gut microbiota. It is well documented that saturated fats contribute to obesity not only via increased caloric input but also through enhanced overflow of dietary fats to the distal intestine, particularly by upregulation of lipid-metabolism genes [128]. At the same time, these changes were observed to reduce microbial diversity and increase the Firmicutes/Bacteroidetes ratio. Further, at the genus level, saturated fats have been shown to relatively increase gram-negative bacterium including *Fusobacterium*, *Tyzzerella*, *Anaerotruncus*, *Lachnospiraceae*, *Eisenbergiella* and *Escherichia* [126,127,129]. In turn, the production of lipopolysaccharides by these gut bacteria promotes a pro-inflammatory state leading to metabolic endotoxemia and chronic inflammation. Likewise, obesogenic diets trigger adipose tissue inflammation indicated by elevated levels of genes encoding for Mcp1 and F4/80, proteins involved in macrophage infiltration [130]. This is also associated with an increase in the expression of Il-10, an anti-inflammatory cytokine that may represent the body’s compensatory response to chronic metabolic inflammation.

Importantly, states of metabolic endotoxemia and chronic inflammation have been shown to significantly alter satiation regulatory pathways. Specifically, low dose administration of LPS for 6 weeks in a rodent model increased hyperphagic behaviors and reduced CCK-induced satiety and vagal afferent leptin signaling, measured through decreased leptin-induced STAT3 phosphorylation [131]. Similarly, LPS-induced metabolic endotoxemia is shown to induce hyperglycemia via GLP-1 dependent mechanisms [132]. Further, consumption of trans-fats increases expression of hypothalamic cytokines, IL-6, TNF-α and IL-1β, as well as TLR-4 and NF-Kβ signaling pathways promoting deleterious effects on satiation in a rodent model [133]. However, it should be noted that gut microbiota dependent mechanisms may also play a role in the short-term control of food intake, independent of chronic inflammatory states and metabolic endotoxemia [134]. When comparing a standard diet with a high fat diet, the measurement of microbiota derived signals indicated increased anorectic effect only after consumption of a high-fat diet [134]. These findings indicate that gut microbiota are important regulators of satiation signals and appetite even under normal physiology in response to meals of varying fat composition.

### 4.2. Omega-3 Polyunsaturated Fatty Acids

It is well documented that polyunsaturated fatty acids (PUFA), including omega-3 and omega-6 fatty acids, play a crucial role in health and disease prevention, such as cardiovascular health, skeletal muscle metabolism, and diabetes. Although the literature on the impact of isolated PUFA on altering gut microbiota is relatively sparse, recent studies have demonstrated positive trends in microbiota composition following PUFA intake including decreases in the Firmicutes/Bacteroides ratio in humans [135]. More specifically, these trends have shown increased abundances of butyrate producing genera including *Roseburia*, *Eubacterium*, *Lactobacillus* and *Bifidobacterium*, along with decreases in *Veillonella* and *Phascolarctobacterium* [135,136,137]. As such, PUFA may exert a myriad of benefits on satiation signals through increases in SCFA as described in much detail throughout this review.

Further, omega-3 PUFAs can also affect health outcomes through the modulation of the hypothalamic inflammation pathways that are early factors in the progression of obesity. For example, docosahexaenoic acid, an omega-3 PUFA, decreased energy intake and weight gain in high-fat diet-induced hypothalamic inflammation and increased central leptin sensitivity in a mouse model [138]. This effect was shown through decreased SOSC3 as well as enhancing signaling via the Jak2-Akt pathway activity. Increased Akt phosphorylation after leptin-induced JAK2-STAT3 activation serves as a marker for increased leptin activity [139]. Further, PUFA has also been shown to exert significant effects on ghrelin and PYY. For example, 7-day consumption of omega-3 PUFA promoted decreased fasting ghrelin and increased fasting peptide YY [85]. Additionally, diets rich in unsaturated fatty acids are also associated with favorable changes in central neuropeptides controlling food intake. As such, the introduction of omega-3 and omega-9 fatty acids in diet-induced obesity reduced hypothalamic expression of NPY and MCH while increasing expression of POMC and CART in a mouse model [140]. Taken together, these findings show contrasting effects that PUFA can exert on satiation signals compared to SFA.

### 4.3. Mediterranean Diet

The Mediterranean diet (MD), composed of minimally processed plant-based foods including fruits, vegetables, fibers, wheats, nuts, seeds, whole-grain cereals and PUFA, has garnered substantial interest due to its anti-inflammatory and antioxidant effects that have been shown to have significant benefits in the treatment of several disease states [141,142,143]. Dietary interventions are responsible for over 50% of the structural variation observed in the gut microbiota [144]. Therefore, consistent adherence to the MD has shown improvements in intestinal barrier integrity, inflammation and insulin sensitivity through gut-microbiota dependent mechanisms. In general, the MD promotes growth of favorable gut microbial species, often contrasting the gut enterotypes observed in studies evaluating the effects of high-fat diets [145]. The food types comprising the MD contain greater amounts of fermentable carbohydrates that can be metabolized by bacterial species into SCFA. At the same time, these fermentable carbohydrates create an environment that supports the growth and relative abundances of commensal gut microbiota. The MD has been shown to increase the overall concentrations of *Lactobacillus* spp., *Bifidobacterium*, *Coprococcus*, *Dorea*, *Eubacterium*, and *Lachnospiraceae* [146]. *Lactobacillus*, in particular, was found to regulate adipocytokines to exert anti-obesity effects on mice that are fed a HFD [147]. Evidence shows that *Lactobacillus* treatment downregulated gene expression of peroxisome proliferator activated receptor gamma (PPAR-γ), TNF-α and fatty acid synthase (FAS). PPAR-γ is a transcription factor expressed in hepatocytes and is closely correlated with adipocyte differentiation processes [148], while FAS is a multi-enzyme protein catalyzing the synthesis of fatty acids [149]. Therefore, dietary interventions such as the MD with increased *Lactobacillus* may reverse PPAR-γ and FAS-mediated adipogenesis that is upregulated in individuals with obesity and its related metabolic diseases.

Further, studies assessing changes in the microbiome in obese individuals after 4 weeks of MD intervention showed increases in *Faecalibacterium prausnitzii* while reducing *Ruminococcus torques* and *Ruminococcus gnavus* [150]. *Faecalibacterium prausnitzii* is a gut bacterial species with important anti-inflammatory effects and significant butyrate producing capacity that has critical implications in metabolic health [151,152]. More specifically, *Faecalibacterium prausnitzii* produces a metabolite called microbial anti-inflammatory molecule that elevated zona-occludens-1, restoring intestinal barrier function. Further, through its production of butyrate, this bacterial species promotes Foxp3 inhibition of the Interleukin-6 (IL-6) signal transducer and activator of transcription 3 (STAT-3) and interleukin-17 (IL-1) pathway [153]. *Roseburia* is another bacterial genus that increases in response to MD adherence [154]. Similar to *Faecalibacterium prausnitzii*, the butyrate producing capacity of *Roseburia* spp. is shown to mediate anti-inflammatory effects to normalize gut homeostasis [155]. For example, *Roseburia intestinalis* reduced serum concentrations of pro-inflammatory cytokines [156]. Additionally, its flagellin inhibits the NLRP3 inflammasome activation in macrophages and associated inflammatory response.

It is well documented that the Mediterranean diet exerts significant anti-inflammatory effects. For example, increases in *Faecalibacterium prausnitzii* and *Roseburia* following MD adherence may decrease inflammatory processes in areas of the brain involved with satiety peptide signaling of which NF-KB/NLRP3 signaling pathways have shown to be strongly associated with hypothalamic inflammation [157]. These changes result in improved insulin sensitivity, weight loss, and decreased circulating serum leptin, after long-term MD feeding in conjunction with exercise, indicating improved leptin sensitivity [158]. These findings were supported by a recent study showing improvements in hyperleptinemia and improved glucoregulation in obese individuals adherent to MD. In obesity, leptin resistance promotes significantly elevated serum leptin levels, which has pro-inflammatory consequences further worsening metabolic endotoxemia in these individuals [159]. As such, through its systemic anti-inflammatory effects, the MD may improve metabolic endotoxemia as well as hyperleptinemia secondary to leptin resistance to restore negative changes induced by HFD.

The introduction of MD in obese individuals with elevated *Bacteroides* reduced *Prevotella* spp. and ameliorated insulin sensitivity [150]. *Bacteroides* spp. and Prevotella *copri*, specifically, have been shown to increase the biosynthetic potential of BCAA, which is a marker of insulin resistance [160]. By contrast, high-fat feeding with high *Prevotella copri* enterotype shows deleterious carnitine-based metabolite production that increases the risk of coronary artery disease and type 2 diabetes mellitus [146]. Similarly, high fat diets have been significantly correlated with elevated *Bacteroides*. *Bacteroides* in adequate amounts can serve a commensal role, however, these bacteria have virulence factors and competitive advantages that allow them to overgrow when gut diversity is low as observed in diet-induced obesity [161]. When in abundance, *Bacteroides* spp. utilize mucin for energy, over degrading the mucin layer [162]. This increases gut permeability [163], as well as susceptibility for endotoxemia-induced chronic inflammation and worsening insulin resistance [164]. Interestingly, these processes may be intricately related to the release of GLP-1 in obese individuals. Hyperglycemic states inhibit the secretion of GLP-1 through generation of oxidative stress and increased protein kinase C 2-beta, which is shown to degrade GLP-1 receptors in endothelial cells [165,166]. Due to its antioxidant effects and anti-hyperglycemic effects, the MD enhances GLP-1 action in endothelial cells as well as improving endothelial function in general as measured through flow-mediated dilatation in the brachial artery. Two recent studies have supported these findings with one reporting that obese T2DM human subjects following the MD for 210 days exhibited statistically significant elevations in fasting GLP-1 levels with lower serum glucose [167], and with the other showing similar increases in GLP-1 and improved insulin sensitivity [168]. Taken together, these findings suggest that the MD can support sustained changes in gut microbiota such as normalizing the amounts of *Bacteroides* and *Prevotella copri* and metabolites like BCAA to improve insulin resistance, thereby enhancing GLP-1 secretion and promoting weight loss.

## 5. Microbiota Intervention and Satiation Signals

Due to the global burden of disease caused by high-fat diet-induced obesity, significant research has been directed at restoring these unfavorable metabolic states. The premise of these therapeutic methods stems from their favorable alterations in the gut microbiota, which, in turn, may exert modulatory effects on satiation signals through local activity or signaling via the microbiota-gut-brain axis. Common microbiota-derived therapeutic interventions that have been used include prebiotics, probiotics and synbiotics. Weight loss surgery (WLS) is also commonly used to treat refractory cases of obesity with post-surgical changes in gut microbiota contributing to favorable effects on satiation signals. These will be discussed in further detail in the following subsections.

### 5.1. Probiotics

The use of probiotics in numerous disease states, including metabolic disorders, has become of considerable interest in recent years. Many of the probiotic supplements described in the literature contain species from genera *Lactobacillus* and *Bifidobacterium* [169], though other gut microbial species are also present. *Lactobacillus* and *Bifidobacterium* exert their benefits through improvements in intestinal barrier permeability, suppression of pro-inflammatory cytokines and preventing growth of harmful bacterial species [170]. As such, introducing species from these two genera limits LPS/TLR-4 mediated endotoxemia and reduces chronic inflammatory states induced by dietary fats [171]. Further, *Lactobacillus* and *Bifidobacterium* probiotics are shown to increase SCFA by promoting microbial diversity, specifically of species that contribute to SCFA production [172]. As described throughout this review, SCFA exert a myriad of benefits to the host including the expression of important nutrient sensing receptors, potentiation of satiety signals and modulation of gut peptides, all of which are compromised by a HFD [47,105]. Interestingly, *Lactobacillus* and *Bifidobacterium* are also shown to release low weight antioxidants that mitigate stress from reactive oxygen species [173], further demonstrating the potential roles that these probiotics may have in mitigating the effects of diet-induced obesity.

Several studies have shown the ability of probiotics to restore the unfavorable gut microbial changes secondary to HFD-induced obesity [174] (Table 1). For example, probiotic introduction in HFD-fed mice increased microbial diversity and species that are negatively associated with the onset of obesity such as *Lactobacillus*, *Bifidobacterium*, *Akkermansia* and other butyrate-producing bacterial species [174]. *Akkermansia* spp., in particular, exerts unique benefits including reversal of some of the negative consequences of HFD-induced obesity [175]. More specifically, *Akkermansia* treatment increased gut peptides and lowered adiposity, metabolic endotoxemia and inflammation of adipose tissue. This study identified increases in intestinal endocannabinoids as the potential mechanism by which *Akkermansia* was able to maintain the observed intestinal homeostasis. As indicated earlier, the endocannabinoid system also plays important roles in influencing decision making for food and promoting synaptic plasticity in key brain regions through gut-brain crosstalk [59]. Therefore, probiotic-induced increases in *Akkermansia* spp. not only reverse negative changes in gut health induced by HFD feeding and obesity but also improve neuronal circuits mediating satiety.

Further, probiotics are shown to modulate the neuroendocrine control of appetite by regulating satiety peptide concentrations in human and animal models of obesity [176,191,192]. In a recent study, probiotic administration in obese women increased oxytocin and decreased NPY serum levels compared to placebo groups [176]. The hypothalamic hormone, oxytocin, regulates satiety and feeding behaviors through its anorexigenic effects [193]. In the same study, probiotics improved eating behaviors which coincided with the normalization of oxytocin and NPY levels [176]. Another recent study also supports these findings by showing that a *Lactobacillus plantarum* preparation lowered NPY and leptin mRNA levels compared to control and placebo groups, while also decreasing pro-inflammatory markers [177]. Interestingly, changes in satiety peptides were accompanied by weight gain as well as other overall favorable gut microbiota compositional changes including increased butyrate producing *Akkermansia* and decreased concentrations of *Ruminococcus*, *Dorea* and *Clostridium*. Similarly, other butyrogenic bacteria with probiotic properties, such as *Butyricimonas virosa*, are shown to ameliorate high-fat diet-induced obesity by activation of GLP-1R, improving glucose regulation and upregulated gut barrier tight junction proteins like zonula occludens-1 [178]. This may lead to appetite suppression, weight reduction and metabolic improvement. Similar findings have been reported with *Bifidobacterium longum* in high-fat diet fed mice, which exhibited decreased serum leptin and insulin, with increased GLP-1 after treatment, indicating a strong therapeutic role for butyrate in regulating satiety hormones [179]. These findings are strongly supported by a recent study which showed that a fourteen composite probiotic increased GLP-1 and PYY, through activation of nutrient sensing receptor, GPR41 and GPR-43 [194]. Taken together, these findings provide strong evidence for the role of probiotics in regulating satiety peptides.

### 5.2. Prebiotics

Prebiotics serve as a nutrient source to support the growth of healthy bacteria. Many prebiotics supplements such as oligosaccharides, inulin and polyphenols have been indicated in mitigating the deleterious effects of dietary fats on gut bacteria [180,195,196]. For example, oligofructose-enriched inulin supplementation decreased body fat and body weight in overweight children [180]. The prebiotic supplement increased *Bifidobacterium* spp., while decreasing *Bacteroides* and bile acid concentrations, both of which are characteristic of high-fat induced obesity. In another study with HFD-induced obesity in mice models, oligofructose reduced enterohepatic taurine-conjugated bile acids, which correlate with both obesity and T2DM [197]. This study also assessed beneficial changes secondary to prebiotic introduction of other important gut metabolites from vitamin, fatty acid, steroid and amino acid biosynthesis pathways, which can serve as markers for high-fat-induced obesity. In this regard, gut microbiota derived metabolites ectoine and hippurate are upregulated in oligofructose prebiotic-treated mice. For example, ectoine has positive effects on the integrity of the gut barrier and regulates inflammation via the reduction of key pro-inflammatory cytokines [198], while hippurate is associated with improved metabolic health [199]. Several studies have also shown that prebiotics can reduce ghrelin concentrations in obese individuals [200] and orexigenic neuropeptides, NPY and AgRP, in mice models, providing more insight into their role in normalizing satiety peptides and counteracting hyperphagia [201]. Similarly, inulin-type fructan containing prebiotics support increases in satiety promoting hormones such as GLP-1 [181]. For instance, GLP-1 serum levels were increased in HFD-induced obese murine models after inulin treatment, with decreases in proinflammatory markers including IL-6. The gut microbial composition of these same mice displayed improvements in the Firmicutes/Bacteroidetes ratio, with increases in *Lactobacillus* and other SCFA-producing bacteria like *Lachnospiraceae*. *Desulfovibrio* producing LPS was reduced, which correlates with decreases in inflammatory cytokines like IL-6. Other studies have supported the benefits of inulin by showing improvements in fat oxidation as well as increased SCFA production in as soon as 7 h after consumption by individuals with obesity, though changes in satiety hormone serum concentrations take longer [202]. Importantly, the beneficial effects of inulin on satiety and hypophagia have been shown to be dose dependent [182]. For example, increasing doses of inulin were associated with increases in Bacteroidetes and *Bifidobacterium*, plus CCK transcripts, as well as increased GLP-1 and PYY serum levels. These findings are supported by another study reporting dose-dependent effects of prebiotics, specifically inulin and oligofructose [183]. Again, these changes were associated with increased GLP-1, *Lactobacillus* and *Bifidobacterium*, as well as blunting ghrelin response, thereby reducing energy intake in obese rats. Taken together, these findings suggest that prebiotics have significant effects in improving gut bacterial composition, increasing beneficial gut microbiota-derived metabolites and regulating satiety signals to mitigate abnormalities in energy balance.

### 5.3. Synbiotics

Synbiotic formulations are also indicated in restoring the harmful manifestations of dietary fat-induced obesity and related sequelae like inflammation, hyperlipidemia and insulin resistance [203]. Synbiotics are a combination of prebiotics and probiotics that can potentially be more effective than either of the two alone as they not only introduce beneficial species into the host, but also support their growth. For example, synbiotics containing *Lactobacillus paracasei*, *Bifidobacterium animalis* and β-glucan ameliorate metabolic disturbances associated with diet-induced obesity in animal models [204]. After synbiotic treatment, SCFA production was elevated and bile acid pools were reduced. Further, timing of synbiotic interventions has also been shown to be of importance. Early life synbiotic treatment prevented HFD-induced fat accumulation throughout life by changes in gene expression patterns related to cholesterol synthesis [205]. For example, increases in *Bifidobacterium* were observed at different time points both in early life and through adulthood, indicating that early colonization with this species can have lifetime benefits on metabolic health. A recent study has also shown that symbiotic formulations with *Lactobacillus acidophilus*, *Bifidobacterium lactis*, *Bifidobacterium longum*, *Bifidobacterium bifidum* and a galacto-oligosaccharide mixture improved body composition and biomarkers of obesity in human subjects [23]. The benefits in body composition and weight regulation observed after synbiotic treatment can be attributed to alterations in satiety signals. For example, synbiotic formulation containing seven probiotics strains and fructooligosaccharide improved appetite and weight loss in patients with metabolic syndrome with associated improvements in insulin resistant and increases in GLP-1 and PYY after a 6-week study period [184] (Table 1). Further, synbiotics may reduce hypothalamic inflammation by increasing hypothalamic superoxide dismutase with antioxidant role in brain regions associated with central leptin resistance although serum leptin levels did not change significantly during the study period [185]. However, other studies have shown an improvement in leptin/adiponectin ratio after administration of a *Lactobacillus sakei*, *Leuconostoc kimchi* and allulose synbiotic combination into diet-induced obese mice [186]. These changes were again associated with improvements in inflammatory markers such as IL-6 and IL-1, and associated decreases in fatty acids and triglycerides. Collectively, these studies provide strong evidence for synbiotic formulations in attenuating the effects of HFD-induced obesity by promoting important alterations in satiety hormones.

### 5.4. Weight Loss Surgery

Weight Loss Surgery (WLS) is currently one of the most effective methods to reduce weight for individuals with morbid obesity (BMI > 40) [206]. Specifically, vertical sleeve gastrectomy (VSG) and Roux-en-Y Gastric Bypass (RYGB) are the two common WLS procedures shown to have significant benefits in mechanical food intake restriction, but also for reducing weight and adiposity through characteristic changes in the gut microbiota and satiation signaling in the brain and gut. Though taxonomical modifications in gut microbiota post-WLS are multifactorial and include dietary intake, animal models vs. human studies and surgery type, the shifts toward more favorable enterotypes differ somewhat from those seen after chronic high-fat feeding [207]. For example, in a murine study, VSG was found to restore the negative effects of a chronic high fat diet on gut microbiota composition, with a decreased Firmicutes/Bacteroidetes ratio comparable to normal fed controls even with continued high-fat diet feeding in rats post-VSG [208]. Interestingly, though similarities exist between the two surgical methods [209,210], differing taxonomic changes at the phylum and genus level in VSG and RYGB have been reported. For example, at the phylum level, VSG-treated murine models have demonstrated post-operatively increased Cyanobacteria while the RYGB-operated groups harbored enriched Gammaproteobacteria within their gastrointestinal tract [211]. Further, in VSG, levels of *Eubacterium*, *Blautia* and *Haemophilus* were shown to be elevated in human patients, while *Veillonella*, *Slackia* and *Granucatiella* were increased in RYGB recipients [212]. A recent meta-analysis revealed that the most consistently elevated and associated genera of bacteria from both types of surgery was seen in the genus *Akkermansia* [210]. As mentioned throughout this review, *Akkermansia* has a myriad of benefits for improving gut barrier integrity, satiety peptide activity and increasing SCFA, with recent studies even showing evidence of improvements in food addiction, by modulating mesolimbic dopamine and reducing dopaminergic signaling to the nucleus accumbens [213]. When assessing the predictive value of the pre-operative gut microbial composition that is associated with a better response to VSG, the responders had increased levels of *Bacteroides* while non-responders to expected weight loss exhibited increased *Bacteroides*, *Dorea*, *Ruminococcus* and *Alistipes* [214]. Also, antibiotic administration peri-operatively in mice models of VSG contributed to attenuated weight loss and metabolic improvement, indicating an intricate relationship of gut microbiota [215] in the mechanisms regulating weight loss after surgical interventions. More specifically, in humans, good responders to RYGB as measured by post-surgical estimated weight loss, increased short chain and conjugated primary and secondary bile acids, and did better compared to poorer responders [216]. Bile acids exert their effects through FXR and TGR5, with recent studies showing that hypothalamic TGR5 activation may participate in the anti-obesity effects by activating the sympathetic nervous system to protect from diet-induced obesity and promote an overall negative energy balance [217]. Taken together, there is significant evidence for the role of microbiota and their metabolites in pre-operative, peri-operative and post-operative success in weight loss surgeries.

These changes observed in WLS post-operatively have also been correlated with beneficial alterations in satiation signals as well as through decreases in hypothalamic inflammation. Hypothalamic integrity has been shown to be an indispensable factor for long-term effectiveness and satiety after weight loss surgery [218]. For example, RYGB has been shown to attenuate hypothalamic gliosis, endoplasmic reticulum stress and inflammatory signaling specifically via TLR [187] (Table 1). Evidence also showed upregulation of POMC neurons with associated decreases in the expression of microglia as measured through glial fibrillary acidic protein and IbA1 markers as well as decreased SOCS3 [187]. These changes were hypothesized to be due to RYGB-induced compositional alterations in gut microbiota. Therefore, by reducing LPS-mediated endotoxemia, post-RYGB gut microbiota may contribute to restored anorexigenic effects of central leptin signaling and POMC neuronal activity to promote appetite suppression. In addition to leptin, there has been significant evidence for bariatric surgery-induced changes to serum ghrelin and insulin resistance [219,220]. Importantly, decreased serum ghrelin levels and improved insulin sensitivity have been reported in patients with type 2 diabetes mellitus (T2DM) that underwent RYGB or sleeve gastrectomy. These effects were associated with significant changes in gut microbiota, notably an increase in *Akkermansia*, *Eubacterium*, *Slackia* and *Veillonella*, as well as differentially expressed metabolites post-WLS including decreased BCAA [219]. Similar findings were shown in murine models with high-fat and sugar-induced T2DM that underwent sleeve gastrectomy, with improved insulin sensitivity, ghrelinergic signaling and Firmicutes/Bacteroidetes ratio [220].

Further, enteroendocrine signaling is shown to be enhanced post-prandially in WLS patients, with GLP-1 and PYY consistently shown to be elevated in bariatric surgery patients. A meta-analysis supports these findings by showing increased post-prandial GLP-1 and PYY after sleeve gastrectomy, as well as decreased fasting ghrelin [189] (Table 1). Study findings have suggested that increased GLP-1 following sleeve gastrectomy may be secondary to elevated lithocholic acid, a bile acid produced by gut microbiota [190]. Increases in gut expression of bile acid transporters allow for translocation of lithocholic acid through the gut epithelium. Lithocholic acid mechanistically works through a gut-liver pathway resulting in the production of cholic acid 7-sulfate which agonizes TGR5 to induce GLP-1 secretion [221]. Transplant of gut microbiota after VSG into germ-free animals showed similar findings, providing strong evidence for this mechanistic increase in GLP-1 secondary to changes in microbiota metabolites [190]. Administration of pre- and pro-biotics after RYGB enhances GLP-1, PYY and insulin secretion, further showing the importance of gut microbiota in these processes [222]. Importantly, it has also been shown that increased post-prandial GLP-1 and PYY concentrations in RYGB-operated rodents were associated with an increased preference for a low-fat diet over a high fat diet after 3 weeks [188]. By contrast, GLP-1 and PYY antagonists shifted preference back to high-fat diet in these animals. Taken together, these findings support the role of microbial shifts post-WLS that mediate, in part, changes in satiety peptides, the resultant overall negative energy balance and weight loss. Further research is needed, however, to elucidate more in-depth the stepwise mechanisms by which gut microbiota metabolites contribute to these changes. 

## 6. Conclusions and Perspectives

Substantial evidence supports changes in gut microbiota composition in ways that confer pathophysiological changes in satiation signals, contributing to hyperphagia and obesity. In this review, we focused on how various gut microbial genera resulting from dietary behaviors impact satiety signals, microbiota-derived metabolites and gut-mediated inflammation. These changes in the gut affect satiation signals locally which are also communicated centrally via vagal afferents, inflammatory mediators, enteroendocrine signaling and endocannabinoids. In turn, these mechanisms are intricately interrelated with peripherally mediated signaling via leptin, ghrelin, insulin, CCK, GLP-1 and PYY and centrally mediated neuropeptides NPY, AgRP, POMC and CART to control food intake and regulate energy balance. Since dietary interventions are the main contributors in determining microbial composition [223], we have described the impact that high-fat diets and the MD play in altering gut microbiota and their effect on satiety peptides. We also highlighted therapeutic modalities that mitigate or reverse deleterious changes on satiety signaling observed in HFD-induced obesity such as a probiotic, prebiotics, synbiotics and WLS. 

The presentation of the complexity of the relationship between diet, gut microbiota and the host and its intricate physiological, metabolical, cellular and molecular mechanisms is challenging considering the exponential increase of knowledge in this field. As such, no review, including this one, is able to capture the vast science surrounding the functionality of the human microbiome as a primary determinant of health as well as disease. It is worth noting that the findings discussed in this review, as well as differential outcomes in gut microbial composition, can vary from study to study based on numerous factors including species (i.e human vs. murine studies), time course, sex, etc. that can impact satiety signaling as well as therapeutic interventions to restore deficiencies. Specifically, many of the results presented throughout this review are derived from murine models, while human studies are more limited. Though these studies serve as an appreciable model as humans and murine models share roughly 90% similarities in gut microbiota composition [224], they should be interpreted with caution when generalizing the results from murine models to draw conclusions in humans. Likewise, some of the studies on humans are underpowered, thus limiting their interpretation and ability for making meaningful inferences with the host phenotype and underlying diet-microbiome-mediated pathophysiological mechanisms. Additionally, the heterogeneity in methodological approaches and research protocols of various studies coupled with the limited number of studies examining the interaction between diet-microbiota-satiation signaling and host genetic factors should be given consideration when interpreting the complexity of the subserving mechanisms. 

Notwithstanding, the microbiota altering interventions contribute significantly to the shaping of the gut microbial ecosystem and corresponding mechanisms that control food intake. These collective findings pose the question of whether a specific “microbiota consortia” may exert the most optimal effects for promoting the positive changes on satiation signals to optimize energy balance and reduce hyperphagia. Recent findings have suggested that out of fifteen *Lactobacillus* and six *Bifidobacterium* species studied, *Bifidobacterium longum* as a single strain and the combination of *Lactobacillus gasseri* and *Bifidobacterium lactis* had the most potent GLP-1-inducing effects with improved hypothalamic leptin and POMC gene expression [225]. Next generation probiotic species have also been identified including *Faecalibacterium prausnatzii* and *Akkermansia muciniphila* [226] which are increased significantly after GLP-1 agonist therapy and weight loss [227]. Similarly, *Akkermansia muciniphilia* administered to HFD-induced mice promoted GLP-1 release and ameliorated metabolic disease [96]. Lastly, *Hafnia alvei* has also emerged as a potential strain with significant weight loss effects shown through decreases in AgRP mRNA expression and increased stimulation of PYY and activation of POMC neurons [228,229]. Though there is strong evidence for targeted single/double strain therapy as precision probiotics in treating obesity, it is challenging to establish causality as synergistic and antagonistic effects between the gut microbiome and the host must be considered. As such, this is a difficult task and future studies can build upon these established findings to find optimal therapeutic microbiota consortia to enhance satiety signaling.

## Figures and Tables

**Table 1:** Therapeutic interventions that restore negative effects of chronic high-fat diet feeding, effects on satiety peptides, and improvements in gut microbiota.

Therapeutic Intervention	Study Period	Species Involved/Outcome Measured	Results/Implications	Subject Type	Reference
Mediterranean Diet	1 year	Metabolic Parameters	- Reduced serum leptin, insulin resistance and hgbA1c - Weight loss of >5% in over 33.7% of individuals (21.8% greater than controls) - Improved TG and HDL	Human	[158]
	8 weeks	↑ *Faecalibacterium* ↓ *Ruminococcus*	- Reduced fecal bile acids, systemic inflammation, and total cholesterol	Human	[150]
	210 days	Hunger/Satiety	- Reduced post-prandial glucose release - Biphasic release of GLP-1 and oxyntomodulin - Increased GLP-1 release, peaking after 150 days	Human	[167]
	24 weeks	Metabolic Parameters/Satiety Peptides	- Improved blood fasting glucose, insulin resistance, weight loss, hgbA1c - Increased GLP-1 secretion	Human	[168]
Probiotics	12 weeks	Satiety Peptides/Weight/Eating Behavior	- Decreased serum leptin - Decreased NPY levels and increased oxytocin levels - Improvements in eating behaviors - Significant weight loss measured via BMI	Human	[176]
	12 weeks	↑ *Lactobacillus*, *Alistipes*, *Akkermansia* ↓ *Ruminococcus*, *Dorea*, *Clostridium*	- Heat-killed *Lactobacillus* decreased NPY and leptin, while increasing adipokines - Heat-killed *Lactobacillus* decreased TNF-α and IL-6	Mice	[177]
	6 weeks	Metabolic Parameters/Effect on HFD-induced obesity	- Heat-killed *Butyricimonas* ameliorated HFD-induced weight gain and insulin resistance - GLP-1 receptor and PPAR-α activation was observed - Upregulated zona-occludens in the ileum	Mice	[178]
		Metabolic Parameters/Satiety Peptides	- Selenium-enriched *Bifidobacterium* increased butyrate in feces and lowered pro-inflammatory cytokines - Improved serum and intestinal GLP-1 levels	Mice	[179]
Prebiotics—Inulin/Oligofructose	16 weeks	Metabolic Parameters ↑ *Bifidobacterium* ↓ *Bacteroides*	- Decreased body fat and overall weight - 25% reduction in IL-6 serum level - Decreased amounts of bile acids compared to placebo group	Human	[180]
	12 weeks	Satiety Peptides ↑ *Lachnospiraceae* ↓ *Desulfovibrio*	- Enhanced GLP-1 secretion - Suppressed IL-6 secretion and LPS-producing species - Reduced gluconeogenesis in the liver	Mice	[181]
	21 days	↑ Bacteroidetes, *Bifidobacterium* ↓ *Clostridium*	- Increased plasma peptide YY and GLP-1 - Increased CCK and pro-glucagon transcripts in the cecum and colon - Increased SCFA in the cecum - Findings were dose-dependent	Rats	[182]
	10 weeks	↑ *Lactobacillus*, *Bifidobacterium*	- Plasma ghrelin was attenuated - GLP-1 serum concentrations increased - Energy intake was decreased - Findings were dose dependent	Rats	[183]
Synbiotics	12 weeks	Metabolic Parameters/Satiety Peptides	- GLP-1 and PYY concentrations increased - Fasting blood sugar and insulin levels improved - Subjects lost weight for up to 6 weeks	Human	[184]
	8 weeks	Metabolic and Oxidative Parameters	- Increased hypothalamic SOD and improved oxidative parameters - Improved lipid parameters	Rats	[185]
	12 weeks	Metabolic and Inflammatory Parameters	- Decreased leptin/adiponectin ratio - Lessened IL-6 and IL-1β - Suppressed negative changes of diet-induced obesity	Mice	[186]
Roux-en-Y Gastric Bypass (RYGB)	12 weeks	Satiety/Inflammatory Markers	- Improved leptin sensitivity through decreased hypothalamic inflammation and lower concentrations of SOCS3 - Upregulation of POMC neurons associated with decreased microglia - Decreased hypothalamic TLR-4 and endoplasmic reticulum stress - Antibiotic-induced dysbiosis suppresses leptin effects in these same RYGB subjects	Rats	[187]
	4 to 6 weeks	Satiety Peptides	- Post-prandial GLP-1 and PYY were higher in RYGB rats - Gradual shift in food preference from high fat diet to low fat diet - GLP-1 and PYY antagonist treatment increased preference for high fat diets	Rats	[188]
Vertical Sleeve Gastrectomy (VSG)	Meta Analysis	Satiety Peptides	- Decreased Ghrelin and BMI - Increased GLP-1 and PYY - Unchanged gastric inhibitory peptide	Human	[189]
	6 weeks	Microbiota Metabolites/Satiety Peptides	- Increased bile acid transporter expression due to shift in gut microbiome - Microbiota-metabolite lithocholic acid increases Ca7s, increasing GLP-1 secretion - Transplant of microbiota after VSG into GF mice displays similar effects	Mice	[190]

↑, increase; ↓, decrease.

## References

[1] Endalifer M.L., Diress G. (2020). Epidemiology, Predisposing Factors, Biomarkers, and Prevention Mechanism of Obesity: A Systematic Review. J. Obes..

[2] Zheng Y., Ley S.H., Hu F.B. (2018). Global aetiology and epidemiology of type 2 diabetes mellitus and its complications. Nat. Rev. Endocrinol..

[3] Ataey A., Jafarvand E., Adham D., Moradi-Asl E. (2020). The Relationship Between Obesity, Overweight, and the Human Development Index in World Health Organization Eastern Mediterranean Region Countries. J. Prev. Med. Public. Health.

[4] Golden A. (2021). Obesity’s Impact. Nurs. Clin. N. Am..

[5] Malone J.I., Hansen B.C. (2019). Does obesity cause type 2 diabetes mellitus (T2DM)? Or is it the opposite?. Pediatr. Diabetes.

[6] Cuevas-Sierra A., Ramos-Lopez O., Riezu-Boj J.I., Milagro F.I., Martinez J.A. (2019). Diet, Gut Microbiota, and Obesity: Links with Host Genetics and Epigenetics and Potential Applications. Adv. Nutr..

[7] Gomes A.C., Hoffmann C., Mota J.F. (2018). The human gut microbiota: Metabolism and perspective in obesity. Gut Microbes.

[8] Asadi A., Shadab Mehr N., Mohamadi M.H., Shokri F., Heidary M., Sadeghifard N., Khoshnood S. (2022). Obesity and gut-microbiota-brain axis: A narrative review. J. Clin. Lab. Anal..

[9] Hamamah S., Hajnal A., Covasa M. (2022). Impact of Nutrition, Microbiota Transplant and Weight Loss Surgery on Dopaminergic Alterations in Parkinson’s Disease and Obesity. Int. J. Mol. Sci..

[10] Barber T.M., Valsamakis G., Mastorakos G., Hanson P., Kyrou I., Randeva H.S., Weickert M.O. (2021). Dietary Influences on the Microbiota-Gut-Brain Axis. Int. J. Mol. Sci..

[11] Mann P.E., Huynh K., Widmer G. (2018). Maternal high fat diet and its consequence on the gut microbiome: A rat model. Gut Microbes.

[12] Indiani C., Rizzardi K.F., Castelo P.M., Ferraz L.F.C., Darrieux M., Parisotto T.M. (2018). Childhood Obesity and Firmicutes/Bacteroidetes Ratio in the Gut Microbiota: A Systematic Review. Child. Obes..

[13] Crovesy L., Masterson D., Rosado E.L. (2020). Profile of the gut microbiota of adults with obesity: A systematic review. Eur. J. Clin. Nutr..

[14] Chianese R., Coccurello R., Viggiano A., Scafuro M., Fiore M., Coppola G., Operto F.F., Fasano S., Laye S., Pierantoni R. (2018). Impact of Dietary Fats on Brain Functions. Curr. Neuropharmacol..

[15] Jais A., Paeger L., Sotelo-Hitschfeld T., Bremser S., Prinzensteiner M., Klemm P., Mykytiuk V., Widdershooven P.J.M., Vesting A.J., Grzelka K. (2020). PNOC(ARC) Neurons Promote Hyperphagia and Obesity upon High-Fat-Diet Feeding. Neuron.

[16] Schéle E., Grahnemo L., Anesten F., Hallén A., Bäckhed F., Jansson J.O. (2013). The gut microbiota reduces leptin sensitivity and the expression of the obesity-suppressing neuropeptides proglucagon (Gcg) and brain-derived neurotrophic factor (Bdnf) in the central nervous system. Endocrinology.

[17] Yao H., Fan C., Fan X., Lu Y., Wang Y., Wang R., Tang T., Qi K. (2020). Effects of gut microbiota on leptin expression and body weight are lessened by high-fat diet in mice. Br. J. Nutr..

[18] Gu M., Liu C., Yang T., Zhan M., Cai Z., Chen Y., Chen Q., Wang Z. (2021). High-Fat Diet Induced Gut Microbiota Alterations Associating with Ghrelin/Jak2/Stat3 up-Regulation to Promote Benign Prostatic Hyperplasia Development. Front. Cell Dev. Biol..

[19] Martín M., Rodríguez A., Gómez-Ambrosi J., Ramírez B., Becerril S., Catalán V., López M., Diéguez C., Frühbeck G., Burrell M.A. (2021). Caloric Restriction Prevents Metabolic Dysfunction and the Changes in Hypothalamic Neuropeptides Associated with Obesity Independently of Dietary Fat Content in Rats. Nutrients.

[20] Curone G., Biscarini F., Cotozzolo E., Menchetti L., Dal Bosco A., Riva F., Cremonesi P., Agradi S., Mattioli S., Castiglioni B. (2022). Could Dietary Supplementation with Different Sources of N-3 Polyunsaturated Fatty Acids Modify the Rabbit Gut Microbiota?. Antibiotics.

[21] D’Angelo S., Motti M.L., Meccariello R. (2020). ω-3 and ω-6 Polyunsaturated Fatty Acids, Obesity and Cancer. Nutrients.

[22] Garcia-Mantrana I., Selma-Royo M., Alcantara C., Collado M.C. (2018). Shifts on Gut Microbiota Associated to Mediterranean Diet Adherence and Specific Dietary Intakes on General Adult Population. Front. Microbiol..

[23] Sergeev I.N., Aljutaily T., Walton G., Huarte E. (2020). Effects of Synbiotic Supplement on Human Gut Microbiota, Body Composition and Weight Loss in Obesity. Nutrients.

[24] Furet J.P., Kong L.C., Tap J., Poitou C., Basdevant A., Bouillot J.L., Mariat D., Corthier G., Doré J., Henegar C. (2010). Differential adaptation of human gut microbiota to bariatric surgery-induced weight loss: Links with metabolic and low-grade inflammation markers. Diabetes.

[25] Gupta A., Osadchiy V., Mayer E.A. (2020). Brain-gut-microbiome interactions in obesity and food addiction. Nat. Rev. Gastroenterol. Hepatol..

[26] Clemmensen C., Müller T.D., Woods S.C., Berthoud H.R., Seeley R.J., Tschöp M.H. (2017). Gut-Brain Cross-Talk in Metabolic Control. Cell.

[27] Furness J.B., Callaghan B.P., Rivera L.R., Cho H.J. (2014). The enteric nervous system and gastrointestinal innervation: Integrated local and central control. Adv. Exp. Med. Biol..

[28] Perez-Burgos A., Wang B., Mao Y.K., Mistry B., McVey Neufeld K.A., Bienenstock J., Kunze W. (2013). Psychoactive bacteria Lactobacillus rhamnosus (JB-1) elicits rapid frequency facilitation in vagal afferents. Am. J. Physiol. Gastrointest. Liver Physiol..

[29] Tanida M., Yamano T., Maeda K., Okumura N., Fukushima Y., Nagai K. (2005). Effects of intraduodenal injection of Lactobacillus johnsonii La1 on renal sympathetic nerve activity and blood pressure in urethane-anesthetized rats. Neurosci. Lett..

[30] Loper H., Leinen M., Bassoff L., Sample J., Romero-Ortega M., Gustafson K.J., Taylor D.M., Schiefer M.A. (2021). Both high fat and high carbohydrate diets impair vagus nerve signaling of satiety. Sci. Rep..

[31] Nefti W., Chaumontet C., Fromentin G., Tomé D., Darcel N. (2009). A high-fat diet attenuates the central response to within-meal satiation signals and modifies the receptor expression of vagal afferents in mice. Am. J. Physiol. Regul. Integr. Comp. Physiol..

[32] Sen T., Cawthon C.R., Ihde B.T., Hajnal A., DiLorenzo P.M., de La Serre C.B., Czaja K. (2017). Diet-driven microbiota dysbiosis is associated with vagal remodeling and obesity. Physiol. Behav..

[33] Silva Y.P., Bernardi A., Frozza R.L. (2020). The Role of Short-Chain Fatty Acids from Gut Microbiota in Gut-Brain Communication. Front. Endocrinol..

[34] Siddiqui M.T., Cresci G.A.M. (2021). The Immunomodulatory Functions of Butyrate. J. Inflamm. Res..

[35] Chambers E.S., Preston T., Frost G., Morrison D.J. (2018). Role of Gut Microbiota-Generated Short-Chain Fatty Acids in Metabolic and Cardiovascular Health. Curr. Nutr. Rep..

[36] Braniste V., Al-Asmakh M., Kowal C., Anuar F., Abbaspour A., Tóth M., Korecka A., Bakocevic N., Ng L.G., Kundu P. (2014). The gut microbiota influences blood-brain barrier permeability in mice. Sci. Transl. Med..

[37] Wen J., Ding Y., Wang L., Xiao Y. (2020). Gut microbiome improves postoperative cognitive function by decreasing permeability of the blood-brain barrier in aged mice. Brain Res. Bull..

[38] Li G., Lin J., Zhang C., Gao H., Lu H., Gao X., Zhu R., Li Z., Li M., Liu Z. (2021). Microbiota metabolite butyrate constrains neutrophil functions and ameliorates mucosal inflammation in inflammatory bowel disease. Gut Microbes.

[39] Park M.J., Sohrabji F. (2016). The histone deacetylase inhibitor, sodium butyrate, exhibits neuroprotective effects for ischemic stroke in middle-aged female rats. J. Neuroinflamm..

[40] May A.A., Liu M., Woods S.C., Begg D.P. (2016). CCK increases the transport of insulin into the brain. Physiol. Behav..

[41] Banks W.A., Farr S.A., Salameh T.S., Niehoff M.L., Rhea E.M., Morley J.E., Hanson A.J., Hansen K.M., Craft S. (2018). Triglycerides cross the blood-brain barrier and induce central leptin and insulin receptor resistance. Int. J. Obes..

[42] Miller A.A., Spencer S.J. (2014). Obesity and neuroinflammation: A pathway to cognitive impairment. Brain Behav. Immun..

[43] Kimura I., Ozawa K., Inoue D., Imamura T., Kimura K., Maeda T., Terasawa K., Kashihara D., Hirano K., Tani T. (2013). The gut microbiota suppresses insulin-mediated fat accumulation via the short-chain fatty acid receptor GPR43. Nat. Commun..

[44] Samuel B.S., Shaito A., Motoike T., Rey F.E., Backhed F., Manchester J.K., Hammer R.E., Williams S.C., Crowley J., Yanagisawa M. (2008). Effects of the gut microbiota on host adiposity are modulated by the short-chain fatty-acid binding G protein-coupled receptor, Gpr41. Proc. Natl. Acad. Sci. USA.

[45] Duca F.A., Swartz T.D., Sakar Y., Covasa M. (2012). Increased oral detection, but decreased intestinal signaling for fats in mice lacking gut microbiota. PLoS ONE.

[46] Ang Z., Ding J.L. (2016). GPR41 and GPR43 in Obesity and Inflammation-Protective or Causative?. Front. Immunol..

[47] Lu Y., Fan C., Li P., Lu Y., Chang X., Qi K. (2016). Short Chain Fatty Acids Prevent High-fat-diet-induced Obesity in Mice by Regulating G Protein-coupled Receptors and Gut Microbiota. Sci. Rep..

[48] Koliada A., Syzenko G., Moseiko V., Budovska L., Puchkov K., Perederiy V., Gavalko Y., Dorofeyev A., Romanenko M., Tkach S. (2017). Association between body mass index and Firmicutes/Bacteroidetes ratio in an adult Ukrainian population. BMC Microbiol..

[49] Li H., Page A.J. (2022). Altered Vagal Signaling and Its Pathophysiological Roles in Functional Dyspepsia. Front. Neurosci..

[50] Nighot M., Rawat M., Al-Sadi R., Castillo E.F., Nighot P., Ma T.Y. (2019). Lipopolysaccharide-Induced Increase in Intestinal Permeability Is Mediated by TAK-1 Activation of IKK and MLCK/MYLK Gene. Am. J. Pathol..

[51] Cawthon C.R., de La Serre C.B. (2018). Gut bacteria interaction with vagal afferents. Brain Res..

[52] Cawthon C.R., Kirkland R.A., Pandya S., Brinson N.A., de La Serre C.B. (2020). Non-neuronal crosstalk promotes an inflammatory response in nodose ganglia cultures after exposure to byproducts from gram positive, high-fat-diet-associated gut bacteria. Physiol. Behav..

[53] Yu Y., Park S.J., Beyak M.J. (2019). Inducible nitric oxide synthase-derived nitric oxide reduces vagal satiety signalling in obese mice. J. Physiol..

[54] Chen J., Cheng M., Wang L., Zhang L., Xu D., Cao P., Wang F., Herzog H., Song S., Zhan C. (2020). A Vagal-NTS Neural Pathway that Stimulates Feeding. Curr. Biol..

[55] Park S.J., Yu Y., Zides C.G., Beyak M.J. (2022). Mechanisms of reduced leptin-mediated satiety signaling during obesity. Int. J. Obes..

[56] Ayush E.A., Iwasaki Y., Iwamoto S., Nakabayashi H., Kakei M., Yada T. (2015). Glucagon directly interacts with vagal afferent nodose ganglion neurons to induce Ca^2+^ signaling via glucagon receptors. Biochem. Biophys. Res. Commun..

[57] Lichtman A.H., Blankman J.L., Cravatt B.F. (2010). Endocannabinoid overload. Mol. Pharmacol..

[58] Forte N., Fernández-Rilo A.C., Palomba L., Di Marzo V., Cristino L. (2020). Obesity Affects the Microbiota-Gut-Brain Axis and the Regulation Thereof by Endocannabinoids and Related Mediators. Int. J. Mol. Sci..

[59] Forte N., Boccella S., Tunisi L., Fernández-Rilo A.C., Imperatore R., Iannotti F.A., De Risi M., Iannotta M., Piscitelli F., Capasso R. (2021). Orexin-A and endocannabinoids are involved in obesity-associated alteration of hippocampal neurogenesis, plasticity, and episodic memory in mice. Nat. Commun..

[60] Davidson T.L., Jones S., Roy M., Stevenson R.J. (2019). The Cognitive Control of Eating and Body Weight: It’s More Than What You “Think”. Front. Psychol..

[61] Christie S., O’Rielly R., Li H., Wittert G.A., Page A.J. (2020). High fat diet induced obesity alters endocannabinoid and ghrelin mediated regulation of components of the endocannabinoid system in nodose ganglia. Peptides.

[62] Christie S., O’Rielly R., Li H., Nunez-Salces M., Wittert G.A., Page A.J. (2020). Modulatory effect of methanandamide on gastric vagal afferent satiety signals depends on nutritional status. J. Physiol..

[63] Senin L.L., Al-Massadi O., Folgueira C., Castelao C., Pardo M., Barja-Fernandez S., Roca-Rivada A., Amil M., Crujeiras A.B., Garcia-Caballero T. (2013). The gastric CB1 receptor modulates ghrelin production through the mTOR pathway to regulate food intake. PLoS ONE.

[64] Liu J., Batkai S., Pacher P., Harvey-White J., Wagner J.A., Cravatt B.F., Gao B., Kunos G. (2003). Lipopolysaccharide induces anandamide synthesis in macrophages via CD14/MAPK/phosphoinositide 3-kinase/NF-kappaB independently of platelet-activating factor. J. Biol. Chem..

[65] Moosavi Sohroforouzani A., Shakerian S., Ghanbarzadeh M., Alaei H. (2020). Treadmill exercise improves LPS-induced memory impairments via endocannabinoid receptors and cyclooxygenase enzymes. Behav. Brain Res..

[66] Obradovic M., Sudar-Milovanovic E., Soskic S., Essack M., Arya S., Stewart A.J., Gojobori T., Isenovic E.R. (2021). Leptin and Obesity: Role and Clinical Implication. Front. Endocrinol..

[67] Varela L., Horvath T.L. (2012). Leptin and insulin pathways in POMC and AgRP neurons that modulate energy balance and glucose homeostasis. EMBO Rep..

[68] Liu J., Yang X., Yu S., Zheng R. (2018). The Leptin Resistance. Adv. Exp. Med. Biol..

[69] Shin A.C., MohanKumar S.M.J., Balasubramanian P., Sirivelu M.P., Linning K., Woolcock A., James M., MohanKumar P.S. (2019). Responsiveness of hypothalamo-pituitary-adrenal axis to leptin is impaired in diet-induced obese rats. Nutr. Diabetes.

[70] Liu H., Du T., Li C., Yang G. (2021). STAT3 phosphorylation in central leptin resistance. Nutr. Metab..

[71] Palanivel R., Fullerton M.D., Galic S., Honeyman J., Hewitt K.A., Jorgensen S.B., Steinberg G.R. (2012). Reduced Socs3 expression in adipose tissue protects female mice against obesity-induced insulin resistance. Diabetologia.

[72] Mori H., Hanada R., Hanada T., Aki D., Mashima R., Nishinakamura H., Torisu T., Chien K.R., Yasukawa H., Yoshimura A. (2004). Socs3 deficiency in the brain elevates leptin sensitivity and confers resistance to diet-induced obesity. Nat. Med..

[73] González F., Considine R.V., Abdelhadi O.A., Acton A.J. (2019). Saturated Fat Ingestion Promotes Lipopolysaccharide-Mediated Inflammation and Insulin Resistance in Polycystic Ovary Syndrome. J. Clin. Endocrinol. Metab..

[74] Cho K., Ushiki T., Ishiguro H., Tamura S., Araki M., Suwabe T., Katagiri T., Watanabe M., Fujimoto Y., Ohashi R. (2021). Altered microbiota by a high-fat diet accelerates lethal myeloid hematopoiesis associated with systemic SOCS3 deficiency. iScience.

[75] Mirpuri J., Sotnikov I., Myers L., Denning T.L., Yarovinsky F., Parkos C.A., Denning P.W., Louis N.A. (2012). Lactobacillus rhamnosus (LGG) regulates IL-10 signaling in the developing murine colon through upregulation of the IL-10R2 receptor subunit. PLoS ONE.

[76] Thaler J.P., Yi C.X., Schur E.A., Guyenet S.J., Hwang B.H., Dietrich M.O., Zhao X., Sarruf D.A., Izgur V., Maravilla K.R. (2012). Obesity is associated with hypothalamic injury in rodents and humans. J. Clin. Investig..

[77] Valdearcos M., Robblee M.M., Benjamin D.I., Nomura D.K., Xu A.W., Koliwad S.K. (2014). Microglia dictate the impact of saturated fat consumption on hypothalamic inflammation and neuronal function. Cell Rep..

[78] Mossad O., Batut B., Yilmaz B., Dokalis N., Mezö C., Nent E., Nabavi L.S., Mayer M., Maron F.J.M., Buescher J.M. (2022). Gut microbiota drives age-related oxidative stress and mitochondrial damage in microglia via the metabolite N(6)-carboxymethyllysine. Nat. Neurosci..

[79] Erny D., Hrabě de Angelis A.L., Jaitin D., Wieghofer P., Staszewski O., David E., Keren-Shaul H., Mahlakoiv T., Jakobshagen K., Buch T. (2015). Host microbiota constantly control maturation and function of microglia in the, C.N.S. Nat Neurosci..

[80] Heiss C.N., Mannerås-Holm L., Lee Y.S., Serrano-Lobo J., Håkansson Gladh A., Seeley R.J., Drucker D.J., Bäckhed F., Olofsson L.E. (2021). The gut microbiota regulates hypothalamic inflammation and leptin sensitivity in Western diet-fed mice via a GLP-1R-dependent mechanism. Cell Rep..

[81] Grasset E., Puel A., Charpentier J., Klopp P., Christensen J.E., Lelouvier B., Servant F., Blasco-Baque V., Tercé F., Burcelin R. (2022). Gut microbiota dysbiosis of type 2 diabetic mice impairs the intestinal daily rhythms of GLP-1 sensitivity. Acta Diabetol..

[82] Berkseth K.E., Guyenet S.J., Melhorn S.J., Lee D., Thaler J.P., Schur E.A., Schwartz M.W. (2014). Hypothalamic gliosis associated with high-fat diet feeding is reversible in mice: A combined immunohistochemical and magnetic resonance imaging study. Endocrinology.

[83] Tschöp M., Smiley D.L., Heiman M.L. (2000). Ghrelin induces adiposity in rodents. Nature.

[84] Rahat-Rozenbloom S., Fernandes J., Cheng J., Wolever T.M.S. (2017). Acute increases in serum colonic short-chain fatty acids elicited by inulin do not increase GLP-1 or PYY responses but may reduce ghrelin in lean and overweight humans. Eur. J. Clin. Nutr..

[85] Stevenson J.L., Paton C.M., Cooper J.A. (2017). Hunger and satiety responses to high-fat meals after a high-polyunsaturated fat diet: A randomized trial. Nutrition.

[86] Torres-Fuentes C., Golubeva A.V., Zhdanov A.V., Wallace S., Arboleya S., Papkovsky D.B., Aidy S.E., Ross P., Roy B.L., Stanton C. (2019). Short-chain fatty acids and microbiota metabolites attenuate ghrelin receptor signaling. FASEB J..

[87] Engelstoft M.S., Park W.M., Sakata I., Kristensen L.V., Husted A.S., Osborne-Lawrence S., Piper P.K., Walker A.K., Pedersen M.H., Nøhr M.K. (2013). Seven transmembrane G protein-coupled receptor repertoire of gastric ghrelin cells. Mol. Metab..

[88] Woźniak D., Cichy W., Przysławski J., Drzymała-Czyż S. (2021). The role of microbiota and enteroendocrine cells in maintaining homeostasis in the human digestive tract. Adv. Med. Sci..

[89] Posovszky C., Wabitsch M. (2015). Regulation of appetite, satiation, and body weight by enteroendocrine cells. Part 1: Characteristics of enteroendocrine cells and their capability of weight regulation. Horm. Res. Paediatr..

[90] Lu V.B., Gribble F.M., Reimann F. (2018). Free Fatty Acid Receptors in Enteroendocrine Cells. Endocrinology.

[91] Parker B.J., Wearsch P.A., Veloo A.C.M., Rodriguez-Palacios A. (2020). The Genus Alistipes: Gut Bacteria with Emerging Implications to Inflammation, Cancer, and Mental Health. Front. Immunol..

[92] Farzi A., Ip C.K., Reed F., Enriquez R., Zenz G., Durdevic M., Zhang L., Holzer P., Herzog H. (2021). Lack of peptide YY signaling in mice disturbs gut microbiome composition in response to high-fat diet. FASEB J..

[93] Mazzawi T., Hausken T., El-Salhy M. (2022). Changes in colonic enteroendocrine cells of patients with irritable bowel syndrome following fecal microbiota transplantation. Scand. J. Gastroenterol..

[94] D’Alessio D. (2016). Is GLP-1 a hormone: Whether and When?. J. Diabetes Investig..

[95] Drucker D.J. (2022). GLP-1 physiology informs the pharmacotherapy of obesity. Mol. Metab..

[96] Yoon H.S., Cho C.H., Yun M.S., Jang S.J., You H.J., Kim J.H., Han D., Cha K.H., Moon S.H., Lee K. (2021). Akkermansia muciniphila secretes a glucagon-like peptide-1-inducing protein that improves glucose homeostasis and ameliorates metabolic disease in mice. Nat. Microbiol..

[97] Joffe Y.T., van der Merwe L., Evans J., Collins M., Lambert E.V., September A.V., Goedecke J.H. (2014). Interleukin-6 gene polymorphisms, dietary fat intake, obesity and serum lipid concentrations in black and white South African women. Nutrients.

[98] Ren M., Zhang H., Qi J., Hu A., Jiang Q., Hou Y., Feng Q., Ojo O., Wang X. (2020). An Almond-Based Low Carbohydrate Diet Improves Depression and Glycometabolism in Patients with Type 2 Diabetes through Modulating Gut Microbiota and GLP-1: A Randomized Controlled Trial. Nutrients.

[99] Cawthon C.R., de La Serre C.B. (2021). The critical role of CCK in the regulation of food intake and diet-induced obesity. Peptides.

[100] Covasa M., Grahn J., Ritter R.C. (2000). Reduced hindbrain and enteric neuronal response to intestinal oleate in rats maintained on high-fat diet. Auton. Neurosci..

[101] Kim J.S., Kirkland R.A., Lee S.H., Cawthon C.R., Rzepka K.W., Minaya D.M., de Lartigue G., Czaja K., de La Serre C.B. (2020). Gut microbiota composition modulates inflammation and structure of the vagal afferent pathway. Physiol. Behav..

[102] Muzio L., Viotti A., Martino G. (2021). Microglia in Neuroinflammation and Neurodegeneration: From Understanding to Therapy. Front. Neurosci..

[103] Wang L., Jacobs J.P., Lagishetty V., Yuan P.Q., Wu S.V., Million M., Reeve J.R., Pisegna J.R., Taché Y. (2017). High-protein diet improves sensitivity to cholecystokinin and shifts the cecal microbiome without altering brain inflammation in diet-induced obesity in rats. Am. J. Physiol. Regul. Integr. Comp. Physiol..

[104] Chelakkot C., Choi Y., Kim D.K., Park H.T., Ghim J., Kwon Y., Jeon J., Kim M.S., Jee Y.K., Gho Y.S. (2018). Akkermansia muciniphila-derived extracellular vesicles influence gut permeability through the regulation of tight junctions. Exp. Mol. Med..

[105] Alhabeeb H., AlFaiz A., Kutbi E., AlShahrani D., Alsuhail A., AlRajhi S., Alotaibi N., Alotaibi K., AlAmri S., Alghamdi S. (2021). Gut Hormones in Health and Obesity: The Upcoming Role of Short Chain Fatty Acids. Nutrients.

[106] Lal S., Kirkup A.J., Brunsden A.M., Thompson D.G., Grundy D. (2001). Vagal afferent responses to fatty acids of different chain length in the rat. Am. J. Physiol. Gastrointest. Liver Physiol..

[107] Klingbeil E.A., Cawthon C., Kirkland R., de La Serre C.B. (2019). Potato-Resistant Starch Supplementation Improves Microbiota Dysbiosis, Inflammation, and Gut-Brain Signaling in High Fat-Fed Rats. Nutrients.

[108] Ahn W., Latremouille J., Harris R.B.S. (2022). Leptin receptor-expressing cells in the ventromedial nucleus of the hypothalamus contribute to enhanced CCK-induced satiety following central leptin injection. Am. J. Physiol. Endocrinol. Metab..

[109] Smith M.A., Choudhury A.I., Glegola J.A., Viskaitis P., Irvine E.E., de Campos Silva P.C.C., Khadayate S., Zeilhofer H.U., Withers D.J. (2020). Extrahypothalamic GABAergic nociceptin-expressing neurons regulate AgRP neuron activity to control feeding behavior. J. Clin. Investig..

[110] Krashes M.J., Koda S., Ye C., Rogan S.C., Adams A.C., Cusher D.S., Maratos-Flier E., Roth B.L., Lowell B.B. (2011). Rapid, reversible activation of AgRP neurons drives feeding behavior in mice. J. Clin. Investig..

[111] Jikomes N., Ramesh R.N., Mandelblat-Cerf Y., Andermann M.L. (2016). Preemptive Stimulation of AgRP Neurons in Fed Mice Enables Conditioned Food Seeking under Threat. Curr. Biol..

[112] Douglass J.D., Dorfman M.D., Fasnacht R., Shaffer L.D., Thaler J.P. (2017). Astrocyte IKKβ/NF-κB signaling is required for diet-induced obesity and hypothalamic inflammation. Mol. Metab..

[113] Ullah R., Rauf N., Nabi G., Yi S., Yu-Dong Z., Fu J. (2021). Mechanistic insight into high-fat diet-induced metabolic inflammation in the arcuate nucleus of the hypothalamus. Biomed. Pharmacother..

[114] Olofsson L.E., Unger E.K., Cheung C.C., Xu A.W. (2013). Modulation of AgRP-neuronal function by SOCS3 as an initiating event in diet-induced hypothalamic leptin resistance. Proc. Natl. Acad. Sci. USA.

[115] Hamamah S., Covasa M. (2022). Gut Microbiota Restores Central Neuropeptide Deficits in Germ-Free Mice. Int. J. Mol. Sci..

[116] Frost G., Sleeth M.L., Sahuri-Arisoylu M., Lizarbe B., Cerdan S., Brody L., Anastasovska J., Ghourab S., Hankir M., Zhang S. (2014). The short-chain fatty acid acetate reduces appetite via a central homeostatic mechanism. Nat. Commun..

[117] Breton J., Tirelle P., Hasanat S., Pernot A., L’Huillier C., do Rego J.C., Déchelotte P., Coëffier M., Bindels L.B., Ribet D. (2021). Gut microbiota alteration in a mouse model of Anorexia Nervosa. Clin. Nutr..

[118] Wang T., Yan H., Lu Y., Li X., Wang X., Shan Y., Yi Y., Liu B., Zhou Y., Lü X. (2020). Anti-obesity effect of Lactobacillus rhamnosus LS-8 and Lactobacillus crustorum MN047 on high-fat and high-fructose diet mice base on inflammatory response alleviation and gut microbiota regulation. Eur. J. Nutr..

[119] Hao L., Sheng Z., Potian J., Deak A., Rohowsky-Kochan C., Routh V.H. (2016). Lipopolysaccharide (LPS) and tumor necrosis factor alpha (TNFα) blunt the response of Neuropeptide Y/Agouti-related peptide (NPY/AgRP) glucose inhibited (GI) neurons to decreased glucose. Brain Res..

[120] Hill J.W. (2010). Gene Expression and the Control of Food Intake by Hypothalamic POMC/CART Neurons. Open Neuroendocrinol. J..

[121] Wang Z.W., Zhou Y.T., Kakuma T., Lee Y., Higa M., Kalra S.P., Dube M.G., Kalra P.S., Unger R.H. (1999). Comparing the hypothalamic and extrahypothalamic actions of endogenous hyperleptinemia. Proc. Natl. Acad. Sci. USA.

[122] Srisai D., Gillum M.P., Panaro B.L., Zhang X.M., Kotchabhakdi N., Shulman G.I., Ellacott K.L., Cone R.D. (2011). Characterization of the hyperphagic response to dietary fat in the MC4R knockout mouse. Endocrinology.

[123] Tam J., Szanda G., Drori A., Liu Z., Cinar R., Kashiwaya Y., Reitman M.L., Kunos G. (2017). Peripheral cannabinoid-1 receptor blockade restores hypothalamic leptin signaling. Mol. Metab..

[124] Morello G., Imperatore R., Palomba L., Finelli C., Labruna G., Pasanisi F., Sacchetti L., Buono L., Piscitelli F., Orlando P. (2016). Orexin-A represses satiety-inducing POMC neurons and contributes to obesity via stimulation of endocannabinoid signaling. Proc. Natl. Acad. Sci. USA.

[125] Kreutzer C., Peters S., Schulte D.M., Fangmann D., Türk K., Wolff S., van Eimeren T., Ahrens M., Beckmann J., Schafmayer C. (2017). Hypothalamic Inflammation in Human Obesity Is Mediated by Environmental and Genetic Factors. Diabetes.

[126] Xu A.A., Kennedy L.K., Hoffman K., White D.L., Kanwal F., El-Serag H.B., Petrosino J.F., Jiao L. (2022). Dietary Fatty Acid Intake and the Colonic Gut Microbiota in Humans. Nutrients.

[127] Bailén M., Bressa C., Martínez-López S., González-Soltero R., Montalvo Lominchar M.G., San Juan C., Larrosa M. (2020). Microbiota Features Associated with a High-Fat/Low-Fiber Diet in Healthy Adults. Front. Nutr..

[128] De Wit N., Derrien M., Bosch-Vermeulen H., Oosterink E., Keshtkar S., Duval C., de Vogel-van den Bosch J., Kleerebezem M., Müller M., van der Meer R. (2012). Saturated fat stimulates obesity and hepatic steatosis and affects gut microbiota composition by an enhanced overflow of dietary fat to the distal intestine. Am. J. Physiol. Gastrointest. Liver Physiol..

[129] Ju T., Bourrie B.C.T., Forgie A.J., Pepin D.M., Tollenaar S., Sergi C.M., Willing B.P. (2023). The Gut Commensal Escherichia coli Aggravates High-Fat-Diet-Induced Obesity and Insulin Resistance in Mice. Appl. Environ. Microbiol..

[130] van der Heijden R.A., Sheedfar F., Morrison M.C., Hommelberg P.P., Kor D., Kloosterhuis N.J., Gruben N., Youssef S.A., de Bruin A., Hofker M.H. (2015). High-fat diet induced obesity primes inflammation in adipose tissue prior to liver in C57BL/6j mice. Aging.

[131] De La Serre C.B., de Lartigue G., Raybould H.E. (2015). Chronic exposure to low dose bacterial lipopolysaccharide inhibits leptin signaling in vagal afferent neurons. Physiol. Behav..

[132] Nguyen A.T., Mandard S., Dray C., Deckert V., Valet P., Besnard P., Drucker D.J., Lagrost L., Grober J. (2014). Lipopolysaccharides-mediated increase in glucose-stimulated insulin secretion: Involvement of the GLP-1 pathway. Diabetes.

[133] Pimentel G.D., Lira F.S., Rosa J.C., Oliveira J.L., Losinskas-Hachul A.C., Souza G.I., das Graças T.d.C.M., Santos R.V., de Mello M.T., Tufik S. (2012). Intake of trans fatty acids during gestation and lactation leads to hypothalamic inflammation via TLR4/NFκBp65 signaling in adult offspring. J. Nutr. Biochem..

[134] Ben Fradj S., Nédélec E., Salvi J., Fouesnard M., Huillet M., Pallot G., Cansell C., Sanchez C., Philippe C., Gigot V. (2022). Evidence for Constitutive Microbiota-Dependent Short-Term Control of Food Intake in Mice: Is There a Link with Inflammation, Oxidative Stress, Endotoxemia, and GLP-1?. Antioxid. Redox Signal.

[135] Liu H., Li X., Zhu Y., Huang Y., Zhang Q., Lin S., Fang C., Li L., Lv Y., Mei W. (2022). Effect of Plant-Derived n-3 Polyunsaturated Fatty Acids on Blood Lipids and Gut Microbiota: A Double-Blind Randomized Controlled Trial. Front. Nutr..

[136] Watson H., Mitra S., Croden F.C., Taylor M., Wood H.M., Perry S.L., Spencer J.A., Quirke P., Toogood G.J., Lawton C.L. (2018). A randomised trial of the effect of omega-3 polyunsaturated fatty acid supplements on the human intestinal microbiota. Gut.

[137] Costantini L., Molinari R., Farinon B., Merendino N. (2017). Impact of Omega-3 Fatty Acids on the Gut Microbiota. Int. J. Mol. Sci..

[138] Cheng L., Hu T., Shi H., Chen X., Wang H., Zheng K., Huang X.F., Yu Y. (2020). DHA reduces hypothalamic inflammation and improves central leptin signaling in mice. Life Sci..

[139] Zheng X., Niu S. (2018). Leptin-induced basal Akt phosphorylation and its implication in exercise-mediated improvement of insulin sensitivity. Biochem. Biophys. Res. Commun..

[140] Cintra D.E., Ropelle E.R., Moraes J.C., Pauli J.R., Morari J., Souza C.T., Grimaldi R., Stahl M., Carvalheira J.B., Saad M.J. (2012). Unsaturated fatty acids revert diet-induced hypothalamic inflammation in obesity. PLoS ONE.

[141] Davis C., Bryan J., Hodgson J., Murphy K. (2015). Definition of the Mediterranean Diet; a Literature Review. Nutrients.

[142] Rosato V., Temple N.J., La Vecchia C., Castellan G., Tavani A., Guercio V. (2019). Mediterranean diet and cardiovascular disease: A systematic review and meta-analysis of observational studies. Eur. J. Nutr..

[143] Mentella M.C., Scaldaferri F., Ricci C., Gasbarrini A., Miggiano G.A.D. (2019). Cancer and Mediterranean Diet: A Review. Nutrients.

[144] Leeming E.R., Johnson A.J., Spector T.D., Le Roy C.I. (2019). Effect of Diet on the Gut Microbiota: Rethinking Intervention Duration. Nutrients.

[145] Dahl W.J., Rivero Mendoza D., Lambert J.M. (2020). Diet, nutrients and the microbiome. Prog. Mol. Biol. Transl. Sci..

[146] Newman T.M., Shively C.A., Register T.C., Appt S.E., Yadav H., Colwell R.R., Fanelli B., Dadlani M., Graubics K., Nguyen U.T. (2021). Diet, obesity, and the gut microbiome as determinants modulating metabolic outcomes in a non-human primate model. Microbiome.

[147] Song W., Song C., Li L., Wang T., Hu J., Zhu L., Yue T. (2021). Lactobacillus alleviated obesity induced by high-fat diet in mice. J. Food Sci..

[148] Lee Y.K., Park J.E., Lee M., Hardwick J.P. (2018). Hepatic lipid homeostasis by peroxisome proliferator-activated receptor gamma 2. Liver Res..

[149] Jiang H., Gan T., Zhang J., Ma Q., Liang Y., Zhao Y. (2019). The Structures and Bioactivities of Fatty Acid Synthase Inhibitors. Curr. Med. Chem..

[150] Meslier V., Laiola M., Roager H.M., De Filippis F., Roume H., Quinquis B., Giacco R., Mennella I., Ferracane R., Pons N. (2020). Mediterranean diet intervention in overweight and obese subjects lowers plasma cholesterol and causes changes in the gut microbiome and metabolome independently of energy intake. Gut.

[151] Xu J., Liang R., Zhang W., Tian K., Li J., Chen X., Yu T., Chen Q. (2020). Faecalibacterium prausnitzii-derived microbial anti-inflammatory molecule regulates intestinal integrity in diabetes mellitus mice via modulating tight junction protein expression. J. Diabetes.

[152] Maioli T.U., Borras-Nogues E., Torres L., Barbosa S.C., Martins V.D., Langella P., Azevedo V.A., Chatel J.M. (2021). Possible Benefits of Faecalibacterium prausnitzii for Obesity-Associated Gut Disorders. Front. Pharmacol..

[153] Zhou L., Zhang M., Wang Y., Dorfman R.G., Liu H., Yu T., Chen X., Tang D., Xu L., Yin Y. (2018). Faecalibacterium prausnitzii Produces Butyrate to Maintain Th17/Treg Balance and to Ameliorate Colorectal Colitis by Inhibiting Histone Deacetylase 1. Inflamm. Bowel Dis..

[154] Rusch C., Beke M., Tucciarone L., Nieves C., Ukhanova M., Tagliamonte M.S., Mai V., Suh J.H., Wang Y., Chiu S. (2021). Mediterranean Diet Adherence in People with Parkinson’s Disease Reduces Constipation Symptoms and Changes Fecal Microbiota after a 5-Week Single-Arm Pilot Study. Front. Neurol..

[155] Nie K., Ma K., Luo W., Shen Z., Yang Z., Xiao M., Tong T., Yang Y., Wang X. (2021). Roseburia intestinalis: A Beneficial Gut Organism from the Discoveries in Genus and Species. Front. Cell. Infect. Microbiol..

[156] Wu X., Pan S., Luo W., Shen Z., Meng X., Xiao M., Tan B., Nie K., Tong T., Wang X. (2020). Roseburia intestinalis-derived flagellin ameliorates colitis by targeting miR-223-3p-mediated activation of NLRP3 inflammasome and pyroptosis. Mol. Med. Rep..

[157] Gan R., Liu H., Wu S., Huang R., Tang Z., Zhang N., Hu L. (2023). Curcumin Alleviates Arsenic Trioxide-Induced Inflammation and Pyroptosis via the NF-κB/NLRP3 Signaling Pathway in the Hypothalamus of Ducks. Biol. Trace Elem. Res..

[158] Salas-Salvadó J., Díaz-López A., Ruiz-Canela M., Basora J., Fitó M., Corella D., Serra-Majem L., Wärnberg J., Romaguera D., Estruch R. (2019). Effect of a Lifestyle Intervention Program with Energy-Restricted Mediterranean Diet and Exercise on Weight Loss and Cardiovascular Risk Factors: One-Year Results of the PREDIMED-Plus Trial. Diabetes Care.

[159] Pérez-Pérez A., Sánchez-Jiménez F., Vilariño-García T., Sánchez-Margalet V. (2020). Role of Leptin in Inflammation and Vice Versa. Int. J. Mol. Sci..

[160] Pedersen H.K., Gudmundsdottir V., Nielsen H.B., Hyotylainen T., Nielsen T., Jensen B.A., Forslund K., Hildebrand F., Prifti E., Falony G. (2016). Human gut microbes impact host serum metabolome and insulin sensitivity. Nature.

[161] Zafar H., Saier M.H. (2021). Gut Bacteroides species in health and disease. Gut Microbes.

[162] Chen L., Wang J., Yi J., Liu Y., Yu Z., Chen S., Liu X. (2021). Increased mucin-degrading bacteria by high protein diet leads to thinner mucus layer and aggravates experimental colitis. J. Gastroenterol. Hepatol..

[163] Pellizoni F.P., Leite A.Z., Rodrigues N.C., Ubaiz M.J., Gonzaga M.I., Takaoka N.N.C., Mariano V.S., Omori W.P., Pinheiro D.G., Matheucci Junior E. (2021). Detection of Dysbiosis and Increased Intestinal Permeability in Brazilian Patients with Relapsing-Remitting Multiple Sclerosis. Int. J. Environ. Res. Public. Health.

[164] Ding N., Zhang X., Zhang X.D., Jing J., Liu S.S., Mu Y.P., Peng L.L., Yan Y.J., Xiao G.M., Bi X.Y. (2020). Impairment of spermatogenesis and sperm motility by the high-fat diet-induced dysbiosis of gut microbes. Gut.

[165] Mima A., Hiraoka-Yamomoto J., Li Q., Kitada M., Li C., Geraldes P., Matsumoto M., Mizutani K., Park K., Cahill C. (2012). Protective effects of GLP-1 on glomerular endothelium and its inhibition by PKCβ activation in diabetes. Diabetes.

[166] Ceriello A., Esposito K., Testa R., Bonfigli A.R., Marra M., Giugliano D. (2011). The possible protective role of glucagon-like peptide 1 on endothelium during the meal and evidence for an “endothelial resistance” to glucagon-like peptide 1 in diabetes. Diabetes Care.

[167] Di Mauro A., Tuccinardi D., Watanabe M., Del Toro R., Monte L., Giorgino R., Rampa L., Rossini G., Kyanvash S., Soare A. (2021). The Mediterranean diet increases glucagon-like peptide 1 and oxyntomodulin compared with a vegetarian diet in patients with type 2 diabetes: A randomized controlled cross-over trial. Diabetes Metab. Res. Rev..

[168] Papamichou D., Panagiotakos D.B., Holmes E., Koutsakis P., Katsoulotos H., Loo R.L., Itsiopoulos C. (2022). The rationale and design of a Mediterranean diet accompanied by time restricted feeding to optimise the management of type 2 diabetes: The MedDietFast randomised controlled trial. Nutr. Metab. Cardiovasc. Dis..

[169] Zhang C., Zhang Y., Li H., Liu X. (2020). The potential of proteins, hydrolysates and peptides as growth factors for Lactobacillus and Bifidobacterium: Current research and future perspectives. Food Funct..

[170] Zhang X.F., Guan X.X., Tang Y.J., Sun J.F., Wang X.K., Wang W.D., Fan J.M. (2021). Clinical effects and gut microbiota changes of using probiotics, prebiotics or synbiotics in inflammatory bowel disease: A systematic review and meta-analysis. Eur. J. Nutr..

[171] Xue L., He J., Gao N., Lu X., Li M., Wu X., Liu Z., Jin Y., Liu J., Xu J. (2017). Probiotics may delay the progression of nonalcoholic fatty liver disease by restoring the gut microbiota structure and improving intestinal endotoxemia. Sci. Rep..

[172] Nagpal R., Shively C.A., Appt S.A., Register T.C., Michalson K.T., Vitolins M.Z., Yadav H. (2018). Gut Microbiome Composition in Non-human Primates Consuming a Western or Mediterranean Diet. Front. Nutr..

[173] Nowak A., Paliwoda A., Błasiak J. (2019). Anti-proliferative, pro-apoptotic and anti-oxidative activity of Lactobacillus and Bifidobacterium strains: A review of mechanisms and therapeutic perspectives. Crit. Rev. Food Sci. Nutr..

[174] Kong C., Gao R., Yan X., Huang L., Qin H. (2019). Probiotics improve gut microbiota dysbiosis in obese mice fed a high-fat or high-sucrose diet. Nutrition.

[175] Everard A., Belzer C., Geurts L., Ouwerkerk J.P., Druart C., Bindels L.B., Guiot Y., Derrien M., Muccioli G.G., Delzenne N.M. (2013). Cross-talk between Akkermansia muciniphila and intestinal epithelium controls diet-induced obesity. Proc. Natl. Acad. Sci. USA.

[176] Narmaki E., Borazjani M., Ataie-Jafari A., Hariri N., Doost A.H., Qorbani M., Saidpour A. (2022). The combined effects of probiotics and restricted calorie diet on the anthropometric indices, eating behavior, and hormone levels of obese women with food addiction: A randomized clinical trial. Nutr. Neurosci..

[177] Nam Y., Yoon S., Baek J., Kim J.H., Park M., Hwang K., Kim W. (2022). Heat-Killed Lactiplantibacillus plantarum LRCC5314 Mitigates the Effects of Stress-Related Type 2 Diabetes in Mice via Gut Microbiome Modulation. J. Microbiol. Biotechnol..

[178] Lee H., An J., Kim J., Choi D., Song Y., Lee C.K., Kong H., Kim S.B., Kim K. (2022). A Novel Bacterium, Butyricimonas virosa, Preventing HFD-Induced Diabetes and Metabolic Disorders in Mice via GLP-1 Receptor. Front. Microbiol..

[179] Zhao D., Zhu H., Gao F., Qian Z., Mao W., Yin Y., Tan J., Chen D. (2020). Antidiabetic effects of selenium-enriched Bifidobacterium longum DD98 in type 2 diabetes model of mice. Food Funct..

[180] Nicolucci A.C., Hume M.P., Martínez I., Mayengbam S., Walter J., Reimer R.A. (2017). Prebiotics Reduce Body Fat and Alter Intestinal Microbiota in Children Who Are Overweight or With Obesity. Gastroenterology.

[181] Zhang Q., Yu H., Xiao X., Hu L., Xin F., Yu X. (2018). Inulin-type fructan improves diabetic phenotype and gut microbiota profiles in rats. PeerJ.

[182] Singh A., Zapata R.C., Pezeshki A., Reidelberger R.D., Chelikani P.K. (2018). Inulin fiber dose-dependently modulates energy balance, glucose tolerance, gut microbiota, hormones and diet preference in high-fat-fed male rats. J. Nutr. Biochem..

[183] Parnell J.A., Reimer R.A. (2012). Prebiotic fibres dose-dependently increase satiety hormones and alter Bacteroidetes and Firmicutes in lean and obese JCR:LA-cp rats. Br. J. Nutr..

[184] Rabiei S., Hedayati M., Rashidkhani B., Saadat N., Shakerhossini R. (2019). The Effects of Synbiotic Supplementation on Body Mass Index, Metabolic and Inflammatory Biomarkers, and Appetite in Patients with Metabolic Syndrome: A Triple-Blind Randomized Controlled Trial. J. Diet. Suppl..

[185] Hosseinifard E.S., Bavafa-Valenlia K., Saghafi-Asl M., Morshedi M. (2020). Antioxidative and Metabolic Effects of Lactobacillus plantarum, Inulin, and Their Synbiotic on the Hypothalamus and Serum of Healthy Rats. Nutr. Metab. Insights..

[186] Choi B.R., Kwon E.Y., Kim H.J., Choi M.S. (2018). Role of Synbiotics Containing d-Allulose in the Alteration of Body Fat and Hepatic Lipids in Diet-Induced Obese Mice. Nutrients.

[187] Chen J., Haase N., Haange S.B., Sucher R., Münzker J., Jäger E., Schischke K., Seyfried F., von Bergen M., Hankir M.K. (2021). Roux-en-Y gastric bypass contributes to weight loss-independent improvement in hypothalamic inflammation and leptin sensitivity through gut-microglia-neuron-crosstalk. Mol. Metab..

[188] Dischinger U., Corteville C., Otto C., Fassnacht M., Seyfried F., Hankir M.K. (2019). GLP-1 and PYY(3-36) reduce high-fat food preference additively after Roux-en-Y gastric bypass in diet-induced obese rats. Surg. Obes. Relat. Dis..

[189] McCarty T.R., Jirapinyo P., Thompson C.C. (2020). Effect of Sleeve Gastrectomy on Ghrelin, GLP-1, PYY, and GIP Gut Hormones: A Systematic Review and Meta-analysis. Ann Surg..

[190] Chaudhari S.N., Luo J.N., Harris D.A., Aliakbarian H., Yao L., Paik D., Subramaniam R., Adhikari A.A., Vernon A.H., Kiliç A. (2021). A microbial metabolite remodels the gut-liver axis following bariatric surgery. Cell Host Microbe.

[191] Valent D., Arroyo L., Fàbrega E., Font I.F.M., Rodríguez-Palmero M., Moreno-Muñoz J.A., Tibau J., Bassols A. (2020). Effects of a high-fat-diet supplemented with probiotics and ω3-fatty acids on appetite regulatory neuropeptides and neurotransmitters in a pig model. Benef. Microbes.

[192] Gioacchini G., Ciani E., Pessina A., Cecchini C., Silvi S., Rodiles A., Merrifield D.L., Olivotto I., Carnevali O. (2018). Effects of Lactogen 13, a New Probiotic Preparation, on Gut Microbiota and Endocrine Signals Controlling Growth and Appetite of Oreochromis niloticus Juveniles. Microb. Ecol..

[193] Burmester V., Nicholls D., Buckle A., Stanojevic B., Crous-Bou M. (2021). Review of eating disorders and oxytocin receptor polymorphisms. J. Eat. Disord..

[194] Wang Y., Wu Y., Sailike J., Sun X., Abuduwaili N., Tuoliuhan H., Yusufu M., Nabi X.H. (2020). Fourteen composite probiotics alleviate type 2 diabetes through modulating gut microbiota and modifying M1/M2 phenotype macrophage in db/db mice. Pharmacol. Res..

[195] Moorthy M., Sundralingam U., Palanisamy U.D. (2021). Polyphenols as Prebiotics in the Management of High-Fat Diet-Induced Obesity: A Systematic Review of Animal Studies. Foods.

[196] Gurpilhares D.B., Cinelli L.P., Simas N.K., Pessoa A., Sette L.D. (2019). Marine prebiotics: Polysaccharides and oligosaccharides obtained by using microbial enzymes. Food Chem..

[197] Meyer R.K., Bime M.A., Duca F.A. (2022). Small intestinal metabolomics analysis reveals differentially regulated metabolite profiles in obese rats and with prebiotic supplementation. Metabolomics.

[198] Abdel-Aziz H., Wadie W., Abdallah D.M., Lentzen G., Khayyal M.T. (2013). Novel effects of ectoine, a bacteria-derived natural tetrahydropyrimidine, in experimental colitis. Phytomedicine.

[199] Brial F., Chilloux J., Nielsen T., Vieira-Silva S., Falony G., Andrikopoulos P., Olanipekun M., Hoyles L., Djouadi F., Neves A.L. (2021). Human and preclinical studies of the host-gut microbiome co-metabolite hippurate as a marker and mediator of metabolic health. Gut.

[200] Da Silva Borges D., Fernandes R., Thives Mello A., da Silva Fontoura E., Soares Dos Santos A.R., Santos de Moraes Trindade E.B. (2020). Prebiotics may reduce serum concentrations of C-reactive protein and ghrelin in overweight and obese adults: A systematic review and meta-analysis. Nutr. Rev..

[201] Delbès A.S., Castel J., Denis R.G.P., Morel C., Quiñones M., Everard A., Cani P.D., Massiera F., Luquet S.H. (2018). Prebiotics Supplementation Impact on the Reinforcing and Motivational Aspect of Feeding. Front. Endocrinol..

[202] Van der Beek C.M., Canfora E.E., Kip A.M., Gorissen S.H.M., Olde Damink S.W.M., van Eijk H.M., Holst J.J., Blaak E.E., Dejong C.H.C., Lenaerts K. (2018). The prebiotic inulin improves substrate metabolism and promotes short-chain fatty acid production in overweight to obese men. Metabolism.

[203] Chiou W.C., Chang B.H., Tien H.H., Cai Y.L., Fan Y.C., Chen W.J., Chu H.F., Chen Y.H., Huang C. (2021). Synbiotic Intervention with an Adlay-Based Prebiotic and Probiotics Improved Diet-Induced Metabolic Disturbance in Mice by Modulation of the Gut Microbiota. Nutrients.

[204] Ke X., Walker A., Haange S.B., Lagkouvardos I., Liu Y., Schmitt-Kopplin P., von Bergen M., Jehmlich N., He X., Clavel T. (2019). Synbiotic-driven improvement of metabolic disturbances is associated with changes in the gut microbiome in diet-induced obese mice. Mol. Metab..

[205] Mischke M., Arora T., Tims S., Engels E., Sommer N., van Limpt K., Baars A., Oozeer R., Oosting A., Bäckhed F. (2018). Specific synbiotics in early life protect against diet-induced obesity in adult mice. Diabetes Obes. Metab..

[206] Wolfe B.M., Kvach E., Eckel R.H. (2016). Treatment of Obesity: Weight Loss and Bariatric Surgery. Circ. Res..

[207] Rossell J., Brindefalk B., Baena-Fustegueras J.A., Peinado-Onsurbe J., Udekwu K.I. (2020). Diet change affects intestinal microbiota restoration and improves vertical sleeve gastrectomy outcome in diet-induced obese rats. Eur. J. Nutr..

[208] Martin O.A., Grant-Beurmann S., Orellana E.R., Hajnal A., Fraser C.M. (2021). Changes in the Gut Microbiota Following Bariatric Surgery Are Associated with Increased Alcohol Intake in a Female Rat Model. Alcohol. Alcohol..

[209] Paganelli F.L., Luyer M., Hazelbag C.M., Uh H.W., Rogers M.R.C., Adriaans D., Berbers R.M., Hendrickx A.P.A., Viveen M.C., Groot J.A. (2019). Roux-Y Gastric Bypass and Sleeve Gastrectomy directly change gut microbiota composition independent of surgery type. Sci. Rep..

[210] Koutoukidis D.A., Jebb S.A., Zimmerman M., Otunla A., Henry J.A., Ferrey A., Schofield E., Kinton J., Aveyard P., Marchesi J.R. (2022). The association of weight loss with changes in the gut microbiota diversity, composition, and intestinal permeability: A systematic review and meta-analysis. Gut Microbes.

[211] Shao Y., Ding R., Xu B., Hua R., Shen Q., He K., Yao Q. (2017). Alterations of Gut Microbiota after Roux-en-Y Gastric Bypass and Sleeve Gastrectomy in Sprague-Dawley Rats. Obes. Surg..

[212] Sánchez-Alcoholado L., Gutiérrez-Repiso C., Gómez-Pérez A.M., García-Fuentes E., Tinahones F.J., Moreno-Indias I. (2019). Gut microbiota adaptation after weight loss by Roux-en-Y gastric bypass or sleeve gastrectomy bariatric surgeries. Surg. Obes. Relat. Dis..

[213] Hankir M.K., Seyfried F., Miras A.D., Cowley M.A. (2018). Brain Feeding Circuits after Roux-en-Y Gastric Bypass. Trends Endocrinol. Metab..

[214] Gutiérrez-Repiso C., Garrido-Sánchez L., Alcaide-Torres J., Cornejo-Pareja I., Ocaña-Wilhelmi L., García-Fuentes E., Moreno-Indias I., Tinahones F.J. (2022). Predictive Role of Gut Microbiota in Weight Loss Achievement after Bariatric Surgery. J. Am. Coll. Surg..

[215] Jahansouz C., Staley C., Kizy S., Xu H., Hertzel A.V., Coryell J., Singroy S., Hamilton M., DuRand M., Bernlohr D.A. (2019). Antibiotic-induced Disruption of Intestinal Microbiota Contributes to Failure of Vertical Sleeve Gastrectomy. Ann. Surg..

[216] Fries C.M., Haange S.B., Rolle-Kampczyk U., Till A., Lammert M., Grasser L., Medawar E., Dietrich A., Horstmann A., von Bergen M. (2022). Metabolic Profile and Metabolite Analyses in Extreme Weight Responders to Gastric Bypass Surgery. Metabolites.

[217] Castellanos-Jankiewicz A., Guzmán-Quevedo O., Fénelon V.S., Zizzari P., Quarta C., Bellocchio L., Tailleux A., Charton J., Fernandois D., Henricsson M. (2021). Hypothalamic bile acid-TGR5 signaling protects from obesity. Cell Metab..

[218] Dischinger U., Kötzner L., Kovatcheva-Datchary P., Kleinschmidt H., Haas C., Perez J., Presek C., Koschker A.C., Miras A.D., Hankir M.K. (2023). Hypothalamic integrity is necessary for sustained weight loss after bariatric surgery: A prospective, cross-sectional study. Metabolism.

[219] Huang H.H., Lin T.L., Lee W.J., Chen S.C., Lai W.F., Lu C.C., Lai H.C., Chen C.Y. (2022). Impact of Metabolic Surgery on Gut Microbiota and Sera Metabolomic Patterns among Patients with Diabetes. Int. J. Mol. Sci..

[220] Su D.W., Wei W.W., Yao R.R., Yang C.J., Tian H. (2020). Research on sleeve gastrectomy for the treatment of rats with type 2 diabetes mellitus and the regulation of ghrelin and intestinal lesions. Eur. Rev. Med. Pharmacol. Sci..

[221] Chaudhari S.N., Harris D.A., Aliakbarian H., Luo J.N., Henke M.T., Subramaniam R., Vernon A.H., Tavakkoli A., Sheu E.G., Devlin A.S. (2021). Bariatric surgery reveals a gut-restricted TGR5 agonist with anti-diabetic effects. Nat. Chem. Biol..

[222] Calikoglu F., Barbaros U., Uzum A.K., Tutuncu Y., Satman I. (2021). The Metabolic Effects of Pre-probiotic Supplementation after Roux-en-Y Gastric Bypass (RYGB) Surgery: A Prospective, Randomized Controlled Study. Obes. Surg..

[223] Zmora N., Suez J., Elinav E. (2019). You are what you eat: Diet, health and the gut microbiota. Nat. Rev. Gastroenterol. Hepatol..

[224] Park J.C., Im S.H. (2020). Of men in mice: The development and application of a humanized gnotobiotic mouse model for microbiome therapeutics. Exp. Mol. Med..

[225] Alard J., Cudennec B., Boutillier D., Peucelle V., Descat A., Decoin R., Kuylle S., Jablaoui A., Rhimi M., Wolowczuk I. (2021). Multiple Selection Criteria for Probiotic Strains with High Potential for Obesity Management. Nutrients.

[226] Dudík B., Kiňová Sepová H., Greifová G., Bilka F., Bílková A. (2022). Next generation probiotics: An overview of the most promising candidates. Epidemiol. Mikrobiol. Imunol..

[227] Remely M., Hippe B., Zanner J., Aumueller E., Brath H., Haslberger A.G. (2016). Gut Microbiota of Obese, Type 2 Diabetic Individuals is Enriched in Faecalibacterium prausnitzii, Akkermansia muciniphila and Peptostreptococcus anaerobius after Weight Loss. Endocr. Metab. Immune Disord. Drug Targets.

[228] Legrand R., Lucas N., Dominique M., Azhar S., Deroissart C., Le Solliec M.A., Rondeaux J., Nobis S., Guérin C., Léon F. (2020). Commensal Hafnia alvei strain reduces food intake and fat mass in obese mice-a new potential probiotic for appetite and body weight management. Int. J. Obes..

[229] Déchelotte P., Breton J., Trotin-Picolo C., Grube B., Erlenbeck C., Bothe G., Fetissov S.O., Lambert G. (2021). The Probiotic Strain H. alvei HA4597^®^ Improves Weight Loss in Overweight Subjects under Moderate Hypocaloric Diet: A Proof-of-Concept, Multicenter Randomized, Double-Blind Placebo-Controlled Study. Nutrients.

